# Induction of innate immunity and plant growth promotion in tomato unveils the antiviral nature of bacterial endophytes against groundnut bud necrosis virus

**DOI:** 10.1128/jvi.01803-24

**Published:** 2024-12-31

**Authors:** R. Sharanya, M. Gayathri, P. Renukadevi, N. Saranya, M. Suganthy, S. Varanavasiappan, Amalendu Ghosh, S. Nakkeeran

**Affiliations:** 1 Department of Plant Pathology, Centre for Plant Protection Studies, Tamil Nadu Agricultural University 29918 , Coimbatore, Tamil Nadu, India; 2 Department of Plant Molecular Biology & Bioinformatics, Centre for Plant Molecular Biology and Biotechnology, Tamil Nadu Agricultural University 29918 , Coimbatore, Tamil Nadu, India; 3 Department of Sustainable Organic Agriculture, Tamil Nadu Agricultural University 29918 , Coimbatore, Tamil Nadu, India; 4 Department of Plant Biotechnology, Centre for Plant Molecular Biology and Biotechnology, Tamil Nadu Agricultural University 29918 , Coimbatore, Tamil Nadu, India; 5 Insect Vector Laboratory, Advanced Centre for Plant Virology, Indian Agricultural Research Institute 28802 , New Delhi, Delhi, India; 6 Agriculture College and Research Institute, Kudumiyanmalai, Pudukottai, Tamil Nadu, India; St. Jude Children's Research Hospital, Memphis, Tennessee, USA

**Keywords:** GBNV, tomato, plant growth promotion, qPCR, DAC-ELISA

## Abstract

Tomato is an important crop worldwide, but groundnut bud necrosis virus (GBNV) often hampers its growth. This study investigates the antiviral potential of bacterial endophytes, including *Brucella melitensis* CNEB54, *Bacillus licheniformis* CNEB4, *Bacillus velezensis* CNEB26, and *Bacillus vallismortis* BAVE5 against GBNV, as well as their ability to enhance immunity and growth in tomato. All four bacterial isolates demonstrated a significant delay in GBNV symptom development 10 days post-inoculation, with disease incidence ranging from 18% to 36% compared to 84% in control. DAC-ELISA results indicated a noteworthy reduction in virus titer (0.32–0.96 OD) in treated tomato plants versus the control (3.26 OD). In addition, qPCR analysis revealed decreased viral copy numbers in plants treated with bacterial endophytes (1.3–3.1 × 10^5^) as against in untreated inoculated control (2.4 × 10^6^). Furthermore, these endophytes upregulated the expression of defense-associated genes, such as *MAPKK1*, *PAL*, *PPO*, *LOX1*, *JAR1*, and *PDF 1.2*. Field experiments with the application of *B. melitensis and B. velezensis* exhibited improved growth, with an average plant height of 123.70 cm, 14.87 flowers per plant, and a fruit weight of 549.3 g per plant, with a disease incidence of 18.1%. In comparison, the untreated control plants only reached a height of 104.73 cm, produced 11.17 flowers per plant, and yielded 267 g of fruit per plant, with a disease incidence of 30.1%. These findings strongly support the use of bacterial endophytes to reduce disease incidence and severity, enhance plant immunity and promote plant growth, resulting in overall crop productivity in sustainable agriculture.

**IMPORTANCE:**

The infection of GBNV in crops such as tomatoes, peanuts, and pulses leads to significant yield loss. Applying insecticides to control vector populations, can limit the spread of viruses carried by these vectors. The present study envisages a novel strategy to combat GBNV, with the help of bacterial endophytes. These bacterial endophytes have tremendously reduced the symptom expression of GBNV, induced the expression of defense genes during the tri-trophic interaction and promoted plant growth in tomatoes under field conditions. Hence, these bacteria are identified to be involved in immunity boosting, viral suppression and growth promotion.

## INTRODUCTION

Tomato is an important vegetable crop and is cultivated globally due to its adaptability to different agro-climatic conditions and high nutritive value. India is the world’s second-largest tomato producer, after China, with an annual production of 20.69 mT (11.12% of global production) (http://www.fao.org/faostat/en/#data/QC). However, biotic and abiotic stresses hinder the cultivation of tomatoes. Among the biotic stress, necrosis disease caused by groundnut bud necrosis virus (GBNV) hampers the yield potential of tomatoes. GBNV is a member of the genus Orthotospovirus, family Tospoviridae, and order Bunyavirales (https://ictv.global/home). Orthotospoviruses are enveloped, isometric virus particles, with a diameter of 80–120 nm. GBNV has a tri-partite genome comprising of ssRNAs, that is, L RNA (8.9 kb negative sense) encoding virus replicase protein of 337 kDa, M RNA (4.8 kb ambisense) encoding two glycoproteins (34 kDa) and movement protein (127 kDa), and S RNA (3.05 kb ambisense) encoding a non-structural small protein (34 kDa) and nucleocapsid protein of 28 KDa (1).

The bud necrosis disease was first identified as the tomato spotted wilt virus in India, in Nilgris, in 1964 (2). However, it was determined in 1992 that bud necrosis disease is caused by a tospovirus distinct from TSWV, known as groundnut bud necrosis virus (GBNV), based on serology and host range studies (1). Three tospoviruses *viz*., GBNV, groundnut yellow spot virus (GYSV), and watermelon bud necrosis virus (WBNV) infect a wide variety of vegetables in India, including tomato, potato, chilli, pepper, and watermelon (1). According to reports, there was a serious GBNV outbreak in several tomato-growing regions of Maharashtra, Karnataka, and Andhra Pradesh between 2003 and 2006, and up to 100% disease incidence was recorded (3). In tomatoes, GBNV appears as necrotic and chlorotic spots on the leaves, petioles, and stem. The young bud dries out and grows more slowly as the infection progresses, which causes the leaves to turn yellow and eventually kills the plant (4). Infected plants produce fruits with smaller concentric chlorotic rings. Dasgupta et al. (5) claimed greater than 80% yield losses in India due to GBNV. GBNV is transmitted by thrips species in a persistent propagative manner (1, 6). Only larval stages effectively acquire the virus and the adults transmit it (7, 8). *Frankliniella schultzei*, *Scirtothrips dorsalis*, and *Thrips palmi* have been reported to transmit GBNV (9).

Viral disease management in plants is cumbersome and our current understanding of the interactions between tospoviruses and thrips is limited. In addition, genetic resistance to a specific virus strain is often quite rare. Transgenic techniques that employ genes derived from viruses can be used to create resistance to viruses. However, the rising concerns in the world for genetically modified foods make them unsuitable. Insecticides to control thrips populations can help to stop the spread of viruses. Nevertheless, an alternate approach is required due to the loss of non-targeted, beneficial microflora and fauna caused by the use of chemicals, as well as the development of chemical resistance in thrips. In these situations, employing beneficial microorganisms that induce host defense mechanisms is a promising way to address this issue. Among the bioagents, *Bacillus* spp. has been widely exploited for the management of various plant diseases. Very few studies have reported the antiviral and antivector efficiency of *Bacillus* species. Earlier studies have demonstrated the use of plant growth-promoting rhizobacteria (PGPR), such as *Pseudomonas fluorescens* that significantly lowered the incidence of tomato spotted wilt virus (TSWV) and also promoted plant growth (10). In addition, it has been reported that PGPR can control other viral diseases in a variety of crops, such as cotton leaf curl (11), tomato mottle virus (12), tobacco mosaic virus (13), cucumber mosaic virus (14) in pepper, and tomato and Tobacco streak virus (TSV) in cotton (15). To understand the innate immune response activated by *Bacillus spp*., a study was conducted by Rajamanickam and Nakkeeran (16) in chilli, Vanthana and coworkers (17, 18) in tomato against GBNV. The transcript level of *WRKY33BB*, *NPR1*, *PAL*, *PPO*, *PDF 1.2*, and *LOX1* genes was significantly upregulated in chilli and tomato plants treated with *Bacillus* spp. respectively. These studies paved the way for the present investigation to evaluate the antiviral efficacy of bacterial endophytes against GBNV in cowpea and tomatoes in both glass house and field conditions.

## MATERIALS AND METHODS

### Virus source and inoculum maintenance

**Fig 1:**
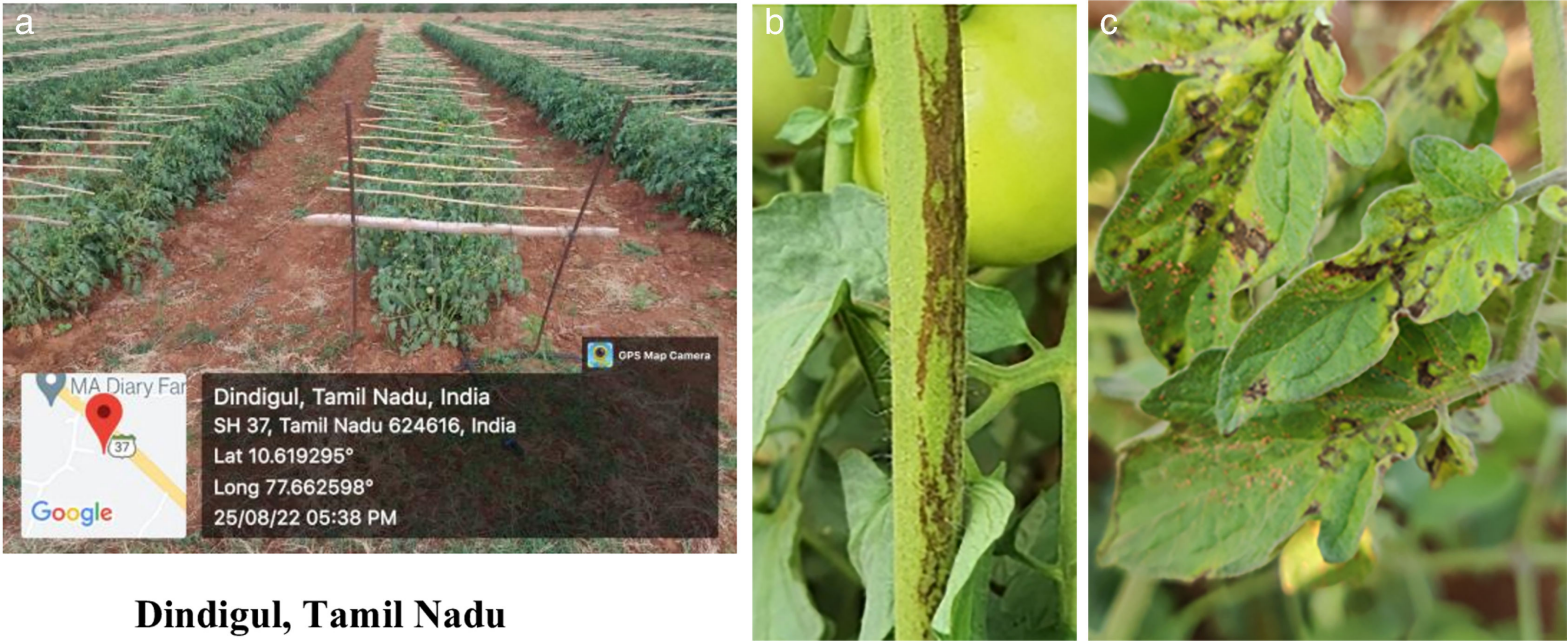
GBNV incidence in the tomato field. (a) GPS data-Dindigul, Tamil Nadu, (b) necrotic rings on leaves, and (c) necrotic streaks on stem.

Tomato plants exhibiting typical necrotic ring spots on leaves and necrotic streaks on the stem, with stunted growth were collected from Karrupadevanpatti, Viralipatti, Thumbhichipalyam, and Koneripatti villages in Dindigul district of Tamil Nadu, India, in 2023 (Fig. 1). This served as a source of inoculum and was maintained in indicator hosts like *Vigna unguiculata* (VBN 3)*, Chenopodium quinoa, Nicotiana tabacum*, and *Datura stramonium* through sap inoculation. Besides, the virus inoculum was also maintained in the main host *Lycopersicum esculentum* (Cv. Saaho). Sap inoculation was performed by macerating the infected tomato leaves in a pre-chilled pestle and mortar with 0.1 M sodium phosphate buffer containing 0.1% β mercaptoethanol (pH 7.2). The indicator plants were dusted with 600-mesh size carborundum powder (Fisher Scientific, USA) uniformly on the primary leaves and the sap was inoculated by gently rubbing the leaf surface. The plants were rinsed with sterile distilled water after 2 minutes and observed for symptom expression in insect-proof glasshouse conditions.

### Detection of GBNV in field-collected samples

The GBNV-infected tomato leaves were subjected to reverse transcription PCR (RT-PCR). The total RNA was extracted through trizol method. cDNA was synthesized using the Takara Bio prime script first-strand cDNA kit. The reaction mixture consists of 4 µL of 5× PrimeScript Buffer, 1 µL each of random primer, 1 µL of oligo dT, 1 µL RNase Inhibitor, and PrimeScript RT, 5 µL of RNA, and 6 µL of nuclease-free water. The synthesized cDNA was amplified using the Nucleocapsid gene (N gene) of GBNV (GBNV NF- 5′ATGTCTAACGT(C/T)AAGCA(A/G)CTC3′ and GBNV NR- 5′TTACAATTCCAGCGAAGGACC3′) to obtain an expected amplicon size of 830 bp. Smart prime ready-to-use master mix was used to perform PCR in a thermocycler (Eppendorf Master cycler, Nexus Gradient, Germany) with Initial denaturation for 2 minutes at 95°C, 40 cycles of denaturation for 30 s at 94°C, annealing for 45 s at 59°C, extension for 1 minute at 72°C, and a final extension for 5 minutes at 72°C. The PCR products were resolved in ethidium bromide-stained, 1% agarose gel, and documented in the gel documentation system (UVP). The PCR product was partially sequenced at M/S Syngenome Lab Pvt. Ltd., Coimbatore. Using Bioedit 2.0, the sequence data were assembled, and a BLAST search in the NCBI databases (https://www.ncbi.nlm.nih.gov) was used for analysis. The sequence was submitted to the NCBI Genbank database, and the accession number was obtained.

### Bacterial endophytes employed to evaluate the antiviral efficacy against GBNV

Totally, 15 bacterial isolates were used for the present study. The bacterial endophytes *viz*., *Bacillus amyloliquefaciens* (MZ485912), *Bacillus glycinifermentans* (MZ485782), *Bacillus altitudinus* (MT326232), *Bacillus mojavensis* (MZ485478), *Bacillus barbaricus* (MT383635), *Bacillus subtilis* (MK259031), *Bacillus albus* (MT120179), *Bacillus siamensis* (MT119641), *Brucella melitensis* (MN022548), *Bacillus licheniformis* (MZ484745), *Bacillus velezensis* (MZ485916), and *Bacillus tequilensis* (MT383662) were obtained from Department of Plant Pathology, TNAU, Coimbatore and the bacterial endophytes *viz*., *Bacillus vallismortis* (MT672715), *Bacillus endophyticus* (KC851835), and *Bacillus aerophilus* (KC851834 ) (Table S1) were sourced from the Department of Agricultural Microbiology, TNAU, Coimbatore. The bacterial inoculum was prepared by inoculation of 24-hour-old bacterial cultures in Luria-Bertani (LB) broth (Sigma-Aldrich) and placed on an orbital shaker at 150 rpm, at 28 ± 2°C for 48 hours. The OD value of the bacterial antagonist was adjusted to 1.5 at A600 nm (10^8^ CFU/mL) in all the experiments.

### *In vitro* screening for the antiviral activity of the bacterial endophytes against GBNV in assay host-cowpea (VBN 3)

In pots filled with sterile potting mixture (Sand: compost: Loamy soil in a 1:2:1 ratio), the seeds of assay host cowpea (variety: VBN 3) were sown and plants were maintained at 28 ± 2°C under glasshouse conditions. The GBNV isolate from Dindigul (DIN) was used in the entire screening study. The efficacy of endophytes was tested as pre (sprayed 24 hours before inoculation of virus) and simultaneous inoculation (Co inoculation of both virus and bacterial isolates) with 1.5% cell suspension culture of different bacterial endophytes. For each treatment, three replications and five plants per replication were maintained in all the treatments including healthy control. Cowpea plants (VBN3) were inoculated with GBNV at the primary leaf stage, in all the treatments except healthy control and were maintained until symptom expression. The reduction in symptom expression was measured in terms of the total number of lesions/leaves compared to the untreated inoculated control. The percent reduction over was calculated by using the formula:


$$Percent\;reduction\;over\;control=\frac{Total\;number\;of\;lesions/leaf\;in\;inoculated\;control-Total\;number\;of\;lesions/leaf\;in\;treatment}{Total\;number\;of\;lesions/leaf\;in\;inoculated\;control}\times 100$$

### Assessment of GBNV titer by DAC-ELISA in endophyte-treated cowpea plants

The GBNV titer in eight effective endophytes treated cowpea plants was detected by measuring the OD value at 405 nm through direct antibody-coated enzyme-linked immunosorbent assay (DAC- ELISA) as per the protocol described by Hobbs et al. in 1987 (19). The dilution of 1:10,000 was used for the polyclonal antisera of GBNV obtained from ICRISAT, Hyderabad, and anti-rabbit IgG (Cat No. #1100180011730, Sigma, Germany) was used as a secondary antibody with dilution of 1:5,000, respectively. The substrate, para-nitrophenyl phosphate at a rate of 1 mg mL^−1^ was used. The symptomatic leaves with local lesions were collected and used for the study. The experiment was performed with three biological replications and three technical replications, for each treatment.

### Screening for the antiviral efficacy of effective bacterial endophytes against GBNV in tomato

Thirty-day-old seedlings of tomato hybrid (Saaho) were purchased from the commercial nursery, transplanted in pots, and maintained at 28 ± 2°C under glasshouse conditions. At fortnightly intervals, the tomato plants were drenched with 2% 19-19-19 (NPK) fertilizer solution. Based on the results of the initial screening and DAC-ELISA in cowpea, the effective bacterial endophytes *viz*., *Brucella melitensis* CNEB54, *Bacillus licheniformis* CNEB4, *Bacillus velezensis* CNEB26, and *Bacillus vallismortis* BAVE5 were screened in tomato as pre (sprayed 24 hours before inoculation of virus) and simultaneous inoculation (Co inoculation of both virus and bacterial isolates) with cell suspension culture of bacterial endophytes comprising 10^8^ CFU/mL against GBNV. Ten days post-transplantation; the plants were inoculated with virus sap as described in the Section “Virus source and inoculum maintenance.” Observation on the number of plants infected, days taken for symptom expression, disease incidence, and delay in symptom expression in each treatment was recorded. Five replications were maintained with five plants per replication.

### Assessment of GBNV titer by DAC-ELISA in tomato plants treated with bacterial endophytes

The virus titer was detected by measuring the OD value at 405 nm through DAC-ELISA in simultaneously inoculated tomato leaves as given in section “Assessment of GBNV titer by DAC-ELISA in endophyte-treated cowpea plants.” The samples were collected on different days post-inoculation (0, 5, 10 DPI) of the virus, and the titer value was assessed. The experiment was conducted with three representative leaf samples per treatment with three technical replications. The OD value was also determined from the newly emerging leaves after 10 DPI.

### Quantification of virus in bacterial endophyte-treated tomato plants by qPCR

The GBNV “N” gene primers (GBNV F-5′GGACCAGATGACTGGACCTTC, GBNV R-5′TCGAAAGCTGCA GGGACAT T3′) designed by Vanthana (17), were used to amplify a 167 bp amplicon through reverse transcriptase quantitative PCR (RT-qPCR) in the BIO-RAD CFX96 manager to quantify the virus. After simultaneous inoculation of endophytes and GBNV inoculum, samples were collected on various days (3rd, 5th, 7th, 9th, and 12th DPI). Five microliters of SYBR Green master mix (KAPA SYBR @FAST for Light Cycler 480, Cat. No. A1250), 1 µL each of forward and reverse primers (10 µM concentration), 2 µL of nuclease-free water, and 1 µL of template cDNA make up the 10 µL reaction volume. The amplification cycle consists of 10 minutes initial denaturation at 95°C and 40 cycles of denaturation for 30 s at 95° C, annealing for 30 s at 60°C, and extension for 30 s at 72°C, followed by standard melting temperature analysis. The absolute quantification method was used to quantify the virus using recombinant plasmid DNA containing the GBNV-CP gene in the pGEMT vector. The copy numbers of GBNV were calculated using the formula:


$$Y\;molecules=X\;g/µL\;DNA\times 6.022\times 10^{23}/(base\;pair\;of\;recombinant\;plasmid\times 660)$$

(The X represents the concentration of plasmid DNA and the Y represents the copy number).

### Real-time PCR analysis on the expression of selected defense-associated genes

The expression of defense-associated genes during tri-trophic interactions was analyzed using RT-qPCR (BIO-RAD CFX manager system). To determine the threshold value, each sample was amplified thrice in triplicate. According to Mascia et al. (2010), the actin gene is stable during virus and viroid interaction with plants. So, in the current investigation, actin served as the internal control. *MAPKK1*, *WRKY33B*, *NPR1*, *PAL*, *PPO*, *JAR1*, *LOX1*, *MYC2*, *PDF* 1.2, and *PR1* were reported as the major defense genes during tri-trophic interaction (18). The expression levels of the above-mentioned genes were studied to understand the mechanism of quenching of GBNV. Table S2 contains the list of the primers used in this study. One pM of each forward and reverse primer were used for each reaction. The fold changes in gene expression were calculated using the formula ΔΔCt = Δ Ct sample – Δ Ct reference, given in reference (20). The delta cycle threshold value is the relative difference between the gene of interest and the reference gene (actin gene). The relative fold changes in the transcript level were represented graphically by converting the ΔΔCT value to 2^−(ΔΔCt)^.

Further heatmap was generated to examine the log 2 fold-change value of defense gene expression in tomatoes under various bacterial treatments using ClustVis software. Color code for different classes: Red represents high expression of a particular gene, white indicates no upregulation of a particular gene, while Blue signifies downregulation of a particular gene. The analysis was carried out totally for 10 genes at an interval of 0, 5, 9, and 12 days.

### Efficacy of bacterial endophytes against GBNV infecting tomato under field condition

The field trial was conducted in 2023 in Anupparpalyam village in the Pollachi region of the Coimbatore district of Tamil Nadu province. Based upon the results of glasshouse studies *Brucella melitensis* CNEB54 and *Bacillus velezensis* CNEB26 were selected for screening under field conditions. For the investigation, the hybrid Saaho was chosen. 30-day-old seedlings were transplanted in the field and the experiment was replicated thrice over an area of 40 m^2^ per replication (approximately 148 plants/40 m^2^). Simultaneously untreated control was also maintained. Individual bacterial strains of OD value 2.5 at A600 nm (10^8^ CFU/mL) (cultures aged 24 hours having) were inoculated into Luria Bertani broth (LB broth) and incubated for 48 hours at room temperature (28±2°C) in an orbital shaker set at 150 rpm. Subsequently, 10% glycerol (10 mL), 10% Tween 20 (10 mL), and 10% polyvinyl pyrrolidone (10 g) from Sigma-Aldrich were combined with the culture broth. To ensure even mixing, the mixture was incubated for 5 minutes at 200 rpm in an orbital shaker. The bacterial cell suspension culture either with 1.5% of *B. melitensis* or *B. velezensis* (4.0 × 10^8^ CFU/mL) separately was blended either with water or in 10% buttermilk base for both soil drenching and foliar spray.

Treatment 1 comprised the cell suspension culture (4 × 10^8^ CFU/mL) of *B. melitensis* (1.5%) delivered in water. Treatment 2 comprised a cell suspension culture (4 × 10^8^ CFU/mL) of *B. velezensis* (1.5%) delivered in water. Treatment 3 consists of the cell suspension culture (4 × 10^8^ CFU/mL) of *B. melitensis* (1.5%) delivered in 10% buttermilk suspended in water. T4 consists of a cell suspension culture (4 × 10^8^ CFU/mL) of *B. velezensis* (1.5%) delivered in 10% buttermilk suspended in water. T5 and T6 pertain to farmer’s practices and untreated control.

Ten days after transplanting, soil drenching was given separately in all four treatments, excluding the control, using the respective treatments at a concentration of 15 mL/L. The same was delivered as a foliar spray at 30, 45, and 60 DAT. Plant growth parameters including, plant height, number of flowers, and fruit weight (g)/plants were recorded in 20 plants from each replication of different treatments. Plants were selected at random and tagged to record observations on various traits. The percent GBNV incidence was recorded periodically and the cumulative GBNV incidence was calculated till harvest.

### Statistical analysis

All the experiments were performed in triplicates and the mean values were evaluated with one-way ANOVA using Duncan’s Multiple Range-Test at 5% significance (21). The normality of the data was checked and then one-way ANOVA was carried out. All the data were statistically analyzed with R software and interpreted. The *P* value was calculated to determine the significance. Each panel represents a mean of six replications in ELISA studies (three biological and three technical replications) and the error bar indicates standard error. In the case of qPCR studies, each panel represents the mean of 6 replications (3 biological and 2 technical replications). Graphs and figures were constructed using Microsoft Excel and R software.

## RESULTS

### Virus inoculum maintenance

Tomato samples exhibiting typical necrotic rings on leaves, necrotic streaks on the stem, with stunting of the whole plant were collected from Karrupadevanpatti, Viralipatti, Thumbhichipalyam, and Koneripatti villages in Dindigul district of Tamil Nadu (Fig. 1). In field conditions, the disease was observed from the young stage to the flowering stage of the crop, with a disease incidence of 35%. The GBNV DIN isolate was maintained in the glasshouse of the Department of Plant Pathology for further studies. The cowpea plants at the primary leaf stage, upon sap inoculation with GBNV exhibited typical chlorotic yellow spots on 4 days post-inoculation (DPI) which later turned into necrotic lesions (Fig. S1). Similarly, necrotic and chlorotic lesions were observed in *Chenopodium quinoa, Datura stramonium,* and *Nicotiana tabacum* within 6–7 DPI (Fig. S1). In the main host tomato, necrotic rings were observed at 8 DPI, and necrotic streaks on the stem were observed after 12 DPI. Furthermore, systemic infection in the form of necrotic rings was also observed in newly emerged leaves, which resulted in the complete drying of plants after 15 DPI (Fig. S1).

### Detection of GBNV in field-collected sample

RNA extracted from the field-collected tomato samples were examined for the presence of GBNV by RT-PCR using specific primers of the nucleocapsid gene (N) of GBNV. The expected amplicon size of ~830 bp was obtained in field-collected samples (Fig. S2). BLAST analysis (http://blast.ncbi.nlm.nih.gov/) revealed the identity of the virus as GBNV. The isolate had a maximum of 100% and a minimum of 98% identity with the nucleocapsid gene sequence of GBNV isolates EF179100 from Andhra Pradesh and OQ473394 from Coimbatore, respectively. The sequence of the isolate is available under accession number OR934931.

### *In vitro* screening for the antiviral activity of the bacterial endophytes against GBNV in the assay host cowpea (VBN 3)

**Fig 2:**
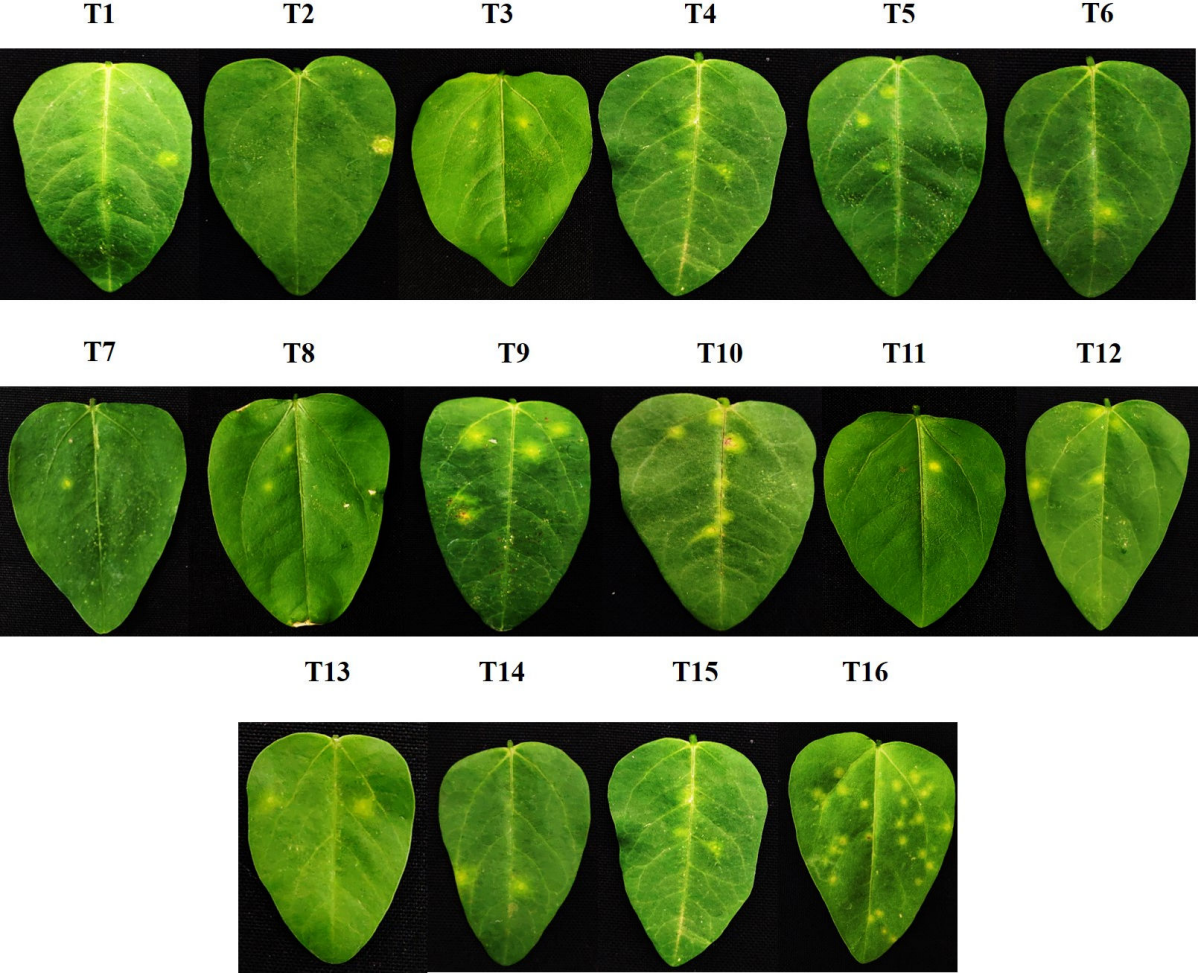
Efficacy of bacterial endophytes against GBNV in cowpea (VBN3) upon simultaneous inoculation.T1-*B. glycinifermentans* CNEB17, T2-*B. altitudinus* BALT, T3-*B. mojavensis* CNEB 14, T4-*B. barbaricus* NPBR1, T5-*B. subtilis* YEBL5, T6-*B. albus* YEBN2, T7-*B. siamensis* YEBN1, T8-*B. amyloliquefaciens* CNEB24, T9-*B. vallismortis BAVE5*, T-10 *B. endophyticus* BA453, T11-*B. melitensis* CNEB54, T12-*B. aerophilus* CNEB3, T13-*B. licheniformis* CNEB4, T14-*B. velezensis* CNEB26, T15*- B. tequilensis* NPN1, T16- untreated inoculated control. Three replications and five plants per replication were maintained for each bacteria.

In both simultaneous and pre-inoculation treatments, all the inoculated leaves expressed typical chlorotic yellow spots on 4 DPI, which later turned into necrotic lesions. Simultaneous inoculation of GBNV and bacterial endophytes proved to be more effective than the pre-inoculation treatment. Among the simultaneous treatments, *Brucella melitensis* was most effective in reducing the lesions to 0.66 lesions/leaves of cowpea, resulting in a 95.39% reduction over untreated inoculated control. This was followed by *B. vallismortis* and *B. licheniformis*, which showed a 92% and 91.2% reduction over untreated inoculated control respectively. *B. velezensis* was also equally effective with 1.86 lesions/leaves, representing an 87.02% reduction over the untreated inoculated control (Table 1; Fig. 2). In the case of pre-inoculation treatment *B. melitensis, B. vallismortis, B. velezensis,* and *B. licheniformis* reduced the symptom expression ranging from 67% to 75% over inoculated control in cowpea (Table 1; Fig. S3).

**TABLE 1:** Efficacy of bacterial endophytes against GBNV in cowpea (VBN 3)^*a*^

S.no	Bacterial endophytes	Total number of lesions/ leaf^*b*^^,^^*c*^			
		Pre inoculation^*d*^	Percent reduction over control^*e*^	Simultaneous inoculation^*f*^	Percent reduction over control^*g*^
1	*Bacillus amyloliquefaciens*	8.46^c^ (16.90)	49.04	1.8^d^ (7.69)	89.16
2	*Bacillus glycinifermentans* CNEB17	5.26^gh^ (13.26)	68.31	0.93^i^ (5.53)	94.40
3	*Bacillus altitudinis* BALT	7.50^d^ (15.88)	54.82	2.0^d^ (8.12)	87.95
4	*Bacillus mojavensis* CNEB14	9.30^b^ (17.74)	43.98	3.36^b^ (10.56)	79.76
5	*Bacillus barbaricus* NPBR1	5.16^h^ (13.13)	68.92	1.36^e^(6.70)	91.81
6	*Bacillus subtilis* YEBL5	7.23^d^ (15.59)	56.45	1.20^gh^ (6.27)	92.77
7	*Bacillus albus* YEBN2	5.90^f^ (14.05)	64.46	1.06^hi^ (6.35)	93.61
8	*Bacillus siamensis*YEBN1	7.53^d^ (15.92)	54.64	1.26^fg^ (6.58)	92.41
9	*Bacillus vallismortis* BAVE5	4.86^hi^ (12.741)	70.72	1.13^hi^ (6.10)	92.11
10	*Bacillus endophyticus* BA453	5.80^fg^ (13.930)	65.06	1.3ef (6.62)	90.71
11	*Bacillus aerophilus* CNEB3	7.8^d^ (16.217)	53.01	3.06^c^ (10.08)	78.64
12	*Brucella melitensis* CNEB54	4.33^i^ (12.014)	73.91	0.66^i^ (4.67)	95.39
13	*Bacillus licheniformis* CNEB4	4.53^i^ (12.289)	72.71	1.26^gh^ (6.45)	91.20
14	*Bacillus velezensis* CNEB26	4.80^hi^ (12.650)	71.08	1.86^d^ (7.83)	87.02
15	*Bacillus tequilensis* NPN1	6.46^e^ (14.729)	61.08	2.86^d^ (9.71)	80.00
16	Inoculated control	17^a^ (23.03)	–^*h*^	14.33^a^ (22.15)	–
17	Uninoculated control	–		–	
	CD	0.623		0.823	
	SE (d)	0.211		0.280	

^*a*^ Data were recorded based on total number of lesions/leaf. Values are the mean of 15 plants (3 replications).

^*b*^ Values in the parentheses are arcsine-transformed values.

^*c*^ In a column, means followed by a common letter are not significantly different at 5% level by DMRT.

^*d*^ Efficacy of bacterial endophytes against GBNV upon pre-inoculation.

^*e*^ Percent reduction over control of pre-inoculation studies.

^*f*^ Efficacy of bacterial endophytes against GBNV upon simultaneous inoculation.

^*g*^ Percent reduction over control of simultaneous inoculation studies.

^*h*^ “–” indicates nill, as percent over reduction is compared with untreated control.

### Assessment of GBNV titer by DAC-ELISA in endophyte-treated cowpea plants

Out of 15 endophytes screened for their effectiveness against GBNV in cowpea, eight highly promising endophytes were identified for their potential antiviral activity. These included *B. glycinifermentans*, *B. albus*, *B. barbaricus*, *B. vallismortis*, *B. melitensis*, *B. endophyticus*, *B. velezensis,* and *B. licheniformis*. Through DAC-ELISA, the virus titer was assessed at A405 nm. The results showed that the untreated inoculated control had an OD value of 1.37, whereas cowpea plants treated with *B. melitensis* (CNEB54) witnessed the lowest OD value of 0.210, followed by 0.361 in *B. velezensis*, 0.427 in *B. licheniformis* (CNEB4), and 0.435 in *B. vallismortis* (BAVE5)-treated plants. The OD value in healthy control was 0.15. This indicated that all eight endophytes were effective in reducing the virus titer, as the OD value was two-fold lower than the inoculated control (Table S3).

### Screening the antiviral efficacy of effective bacterial endophytes against GBNV in tomato

**Fig 3:**
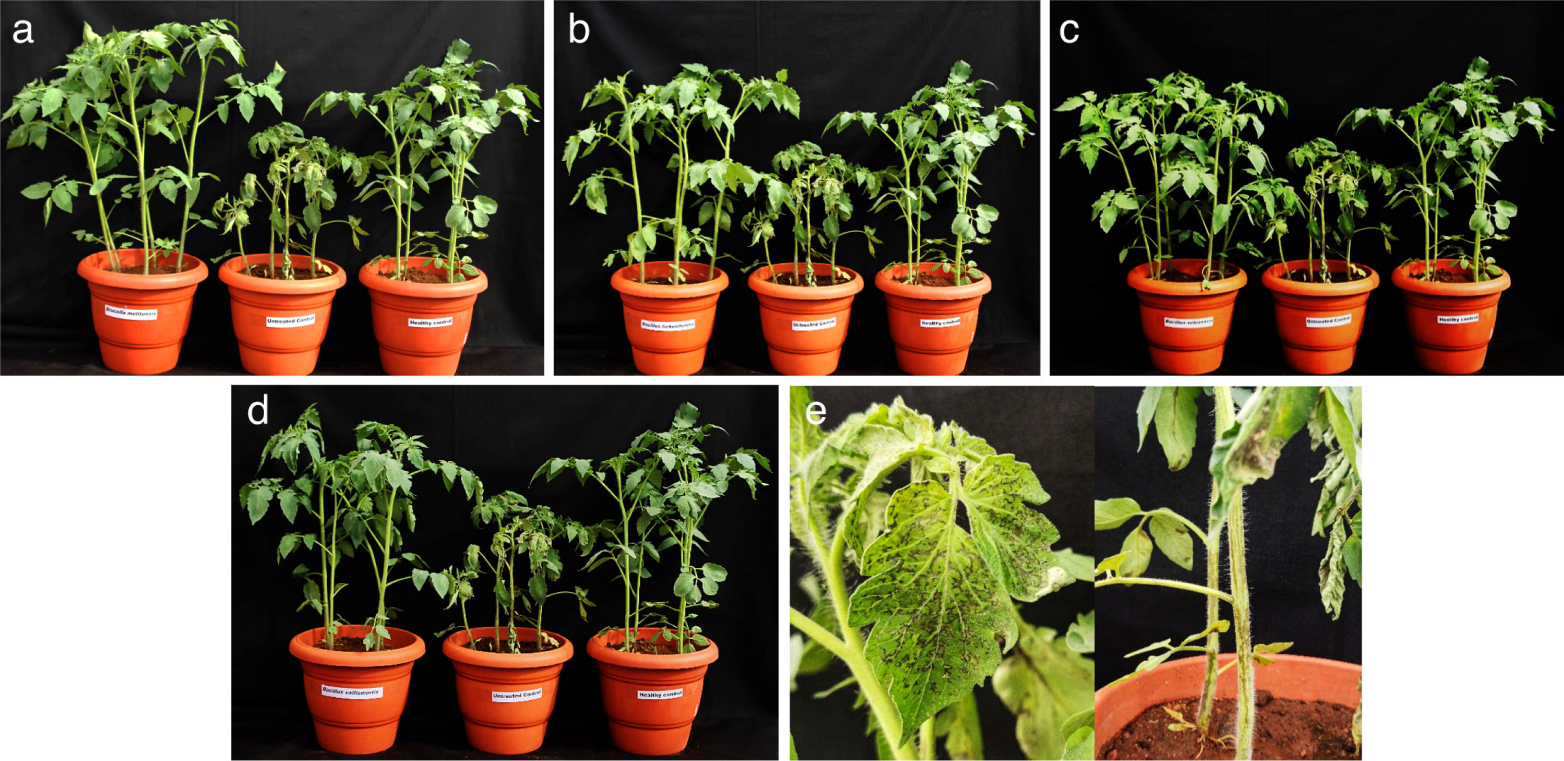
Efficacy of bacterial endophytes against GBNV in tomato (Saaho) upon simultaneous inoculation. (a) *B. melitensis*, (b) *B. licheniformis*, (c) *B. velezensis*, (d) *B. vallismortis*. The T, C, and H represent treated plants, untreated plants, healthy controls respectively. The symptom severity was assessed using symptom severity grades of 0 to 4 (17).

**Fig 4:**
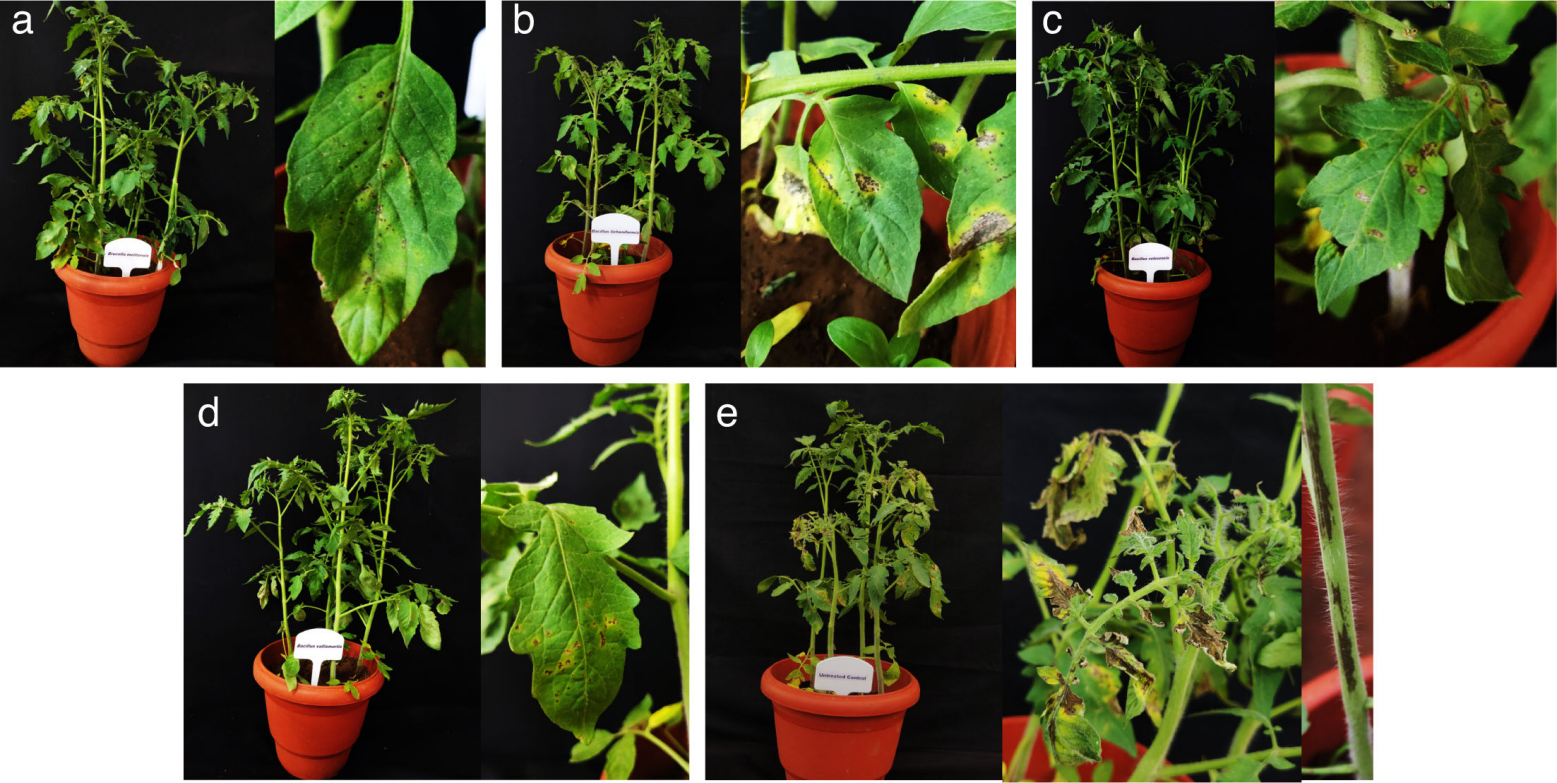
Efficacy of bacterial endophytes against GBNV in tomato (Saaho) upon pre-inoculation. (a) *B. melitensis*, (b) *B. licheniformis*, (c) *B. velezensis*, and (d) *B. vallismortis*. The symptom severity was assessed using symptom severity grades of 0 to 4 (17). The observations on severity were recorded at 12 DPI.

The four effective bacterial endophytes were shortlisted for their antiviral action based on bioassay in cowpea and through DAC-ELISA. They were further evaluated for their effectiveness in the main host tomato. Notably, tomato plants treated with *Brucella melitensis* CNEB54 exhibited only 20% and 8% disease incidence in pre and simultaneous inoculation, while those treated with *B. velezensis* CNEB26 showed 28% and 8% disease incidence in pre and simultaneous inoculation, respectively. By contrast, the untreated inoculated control (GBNV alone) displayed 83% disease incidence (Table 2; Fig. 3 and 4). In addition, a delay in symptom expression was observed in all the bacterial endophyte treated plants. In the case of the tomato plants treated with *Brucella melitensis* CNEB54, symptom expression was observed after 11 DPI, whereas in the untreated control plants, symptoms appeared from the 8th day onward. Furthermore, the symptom severity was assessed using symptom severity grades of 0 to 4 (17). The plants treated with bacterial endophytes showed significantly milder symptoms compared to the severity of 3.04 observed in the inoculated control. The disease severity was a mere 0.20 in the *B. melitensis* treated plants, followed by 0.24 severity in the tomato plants challenged with *B. velezensis*.

**TABLE 2:** Screening of effective bacterial endophytes against GBNV in tomato upon pre and simultaneous inoculation

Treatment	Pre inoculation		Simultaneous inoculation		Days taken for symptom expression
	No. of plants expressed symptom/No. of plants inoculated	Disease severity^*a*^	No. of plants expressed symptom/No. of plants inoculated	Disease^*a*^ severity	
*Brucella melitensis* CNEN54	5/25	0.80	2/25	0.20	11
*Bacillus licheniformis* CNEB4	9/25	0.94	4/25	0.60	12
*Bacillus velezensis* CNEB26	7/25	0.74	2/25	0.24	13
*Bacillus vallismortis* BAVE5	9/25	0.78	3/25	0.44	11
Inoculated control	21/25	3.18	20/25	3.04	9
CD	0.283		0.457		
SE (d)	0.125		0.203		

^*a*^ The disease severity was assessed using symptom severity grades of 0 to 4. The observations on severity were recorded at 12 DPI. Three replications were maintained for each bacteria.

### Assessment of GBNV titer by DAC-ELISA in endophyte-treated tomato plants

**Fig 5:**
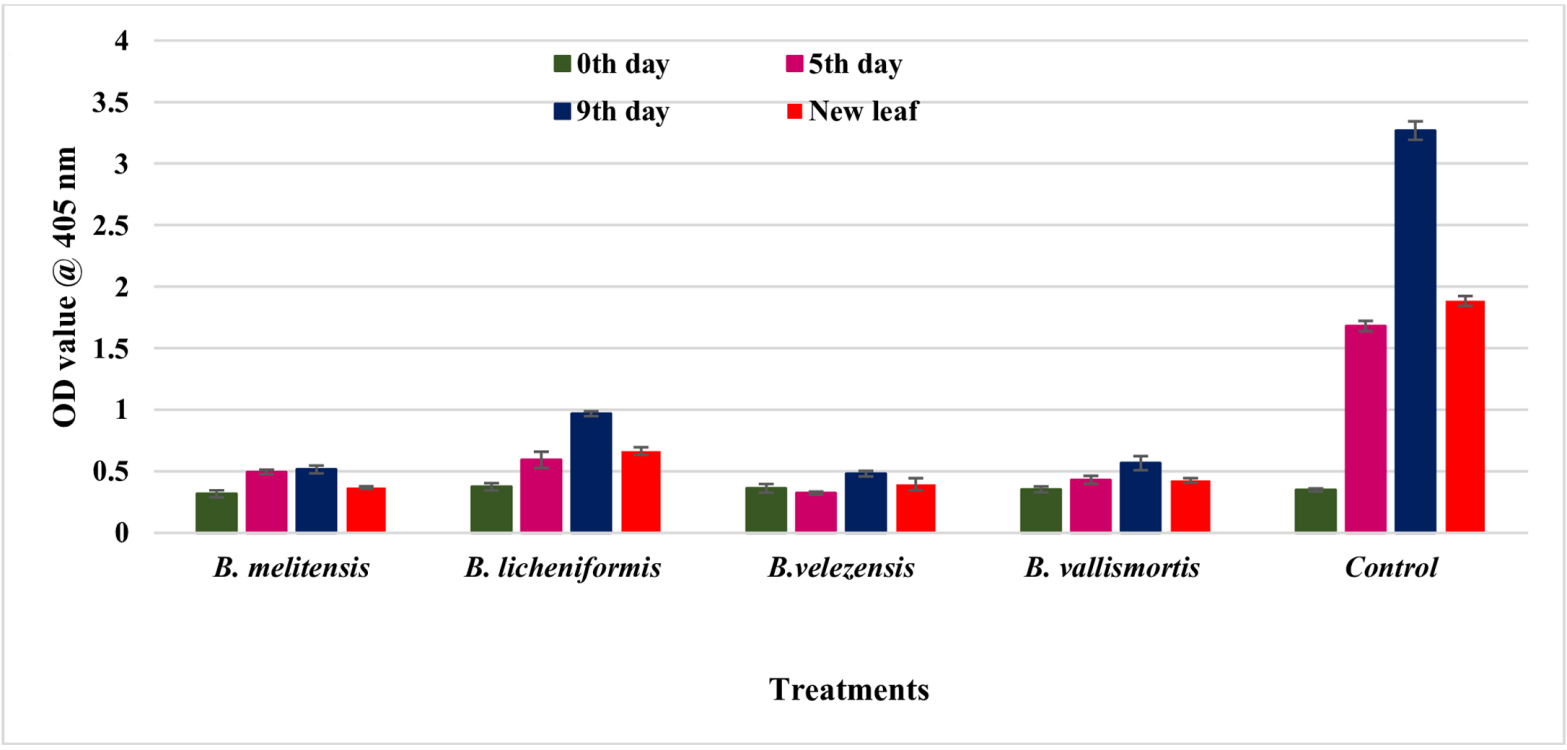
Assessment of GBNV titer in endophyte-treated tomato plants through DAC- ELISA. The experiment was performed with three biological replications and three technical replications, for each treatment. The samples were collected on 0th, 5th, and 10th DPI and even in newly emerged leaves. The green bar indicates the GBNV titer at 0th DPI. The Fuchsia color indicates GBNV titer at the 5th DPI. The blue bar indicates the GBNV titer at the 9th DPI. The red bar indicates the GBNV titer in newly emerged leaves. The error bar indicates the standard error obtained by three replications.

The virus titer was assessed through DAC-ELISA in the effective endophyte-treated tomato plants. In simultaneous inoculation, *B. melitensis-*treated tomato plants recorded the least OD value of 0.317, 0.495, and 0.515 at 0th day, 5th day, and 10th DPI inoculation, respectively. Even in the case of newly emerged leaves, the OD value was least in *B. melitensis* (0.369) treated plants at 10 DPI. Among the four bacterial isolates, the highest OD value was recorded in *B. licheniformis* treated plants at 0th day (0.374), 5th day (0.593), and 10th day (0.968) against inoculated control. In inoculated control, the OD values were 0.349, 1.680, and 3.268, respectively. The newly emerging leaves of the untreated control plants had an OD value of 1.884 (Fig. 5; Table S4).

**Fig 6:**
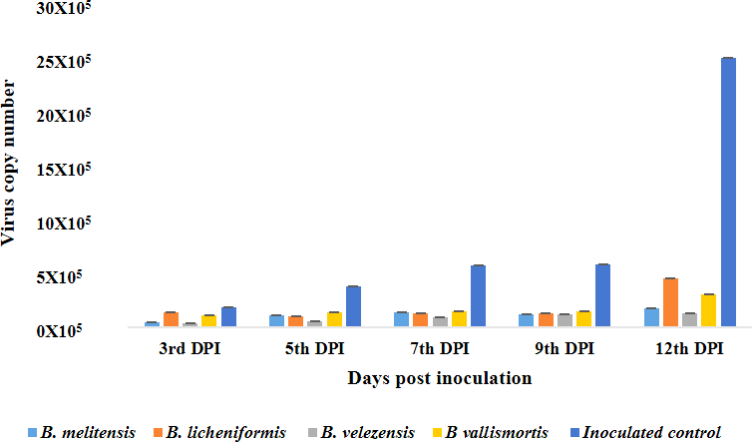
Assessing the copy number of GBNV Nucleocapsid gene (**N**) in bioagents treated tomato plants through real-time PCR. The experiment was performed with three biological replications and two technical replications, for each treatment. Copy number was assessed at different time intervals (0, 3, 5, 9, and 12). The error bar indicates the standard error obtained by three replications.

Furthermore, the virus titer of GBNV was quantified in endophyte-treated plants through qPCR. The virus copy number was determined on different days after inoculation of the virus into tomato (3rd, 5th, 7th, 9th, and 12th DPI). Both bacterial endophytes treated and untreated tomato plants challenged with GBNV showed a consistent increase in virus load from 3 to 12 days following virus inoculation; however, from 5 days post-inoculation, there was a notable reduction in the virus load in the bacterial endophyte treated plants when compared to the untreated inoculated control. At 12 DPI, the lowest copy number of 1.3 × 10^5^ was observed in *B. velezensis* treated plants, compared to 4.5 × 10^5^ in *B. licheniformis* treated plants (Table 3; Fig. 6). The highest copy number of GBNV was recorded in the untreated inoculated control, which was 2.4 × 10^6^ on 12 DPI.

**TABLE 3:** Assessing the copy number of GBNV nucleocapsid gene (N) in bioagent-treated tomato through real-time PCR^*a*^

Bacterial endophytes^*g*^	3rd day^*b*^	5th day^*c*^	7th day^*d*^	9th day^*e*^	12th day^*f*^
*Brucella melitensis*	5.3 × 10^4^	1.1 × 10^5^	1.4 × 10^5^	1.2 × 10^5^	1.7 × 10^5^
*Bacillus licheniformis*	1.4 × 10^5^	1.0 × 10^5^	1.3 × 10^5^	1.3 × 10^5^	4.5 × 10^5^
*Bacillus velezensis*	4.2 × 10^4^	5.6 × 10^4^	9.3 × 10^4^	1.2 × 10^5^	1.3 × 10^5^
*Bacillus vallismortis*	1.4 × 10^5^	1.0 × 10^5^	1.3 × 10^5^	1.3 × 10^5^	3.1 × 10^5^
*inoculated control*	1.8 × 10^5^	3.7 × 10^5^	5.7 × 10^5^	5.8 × 10^5^	2.4 × 10^6^

^*a*^ The experiment was performed with three biological replications and two technical replications, for each treatment.

^*b*^ Copy number of GBNV at 3rd day.

^*c*^ Copy number of GBNV at 5th day.

^*d*^ Copy number of GBNV at 7th day.

^*e*^ Copy number of GBNV at 9th day.

^*f*^ Copy number of GBNV at 12th day.

^*g*^ Bacterial endophytes assessed for their efficacy to reduce the copy number of GBNV.

### Induction of defense gene expression during tri-trophic interaction

#### Role of defense gene in plant immunity

The plant’s immune response involves several receptor-like kinases (RLKs) and receptor-like proteins (RLPs) that have been well described by Tang et al. (2017). When the plant detects pathogen or microbe-associated molecular patterns, these pattern recognition receptors immediately trigger various responses, including the activation of several defense genes. Based on the previous leads on defense gene expression on tri-trophic interaction, the priming effect of the bacterial endophytes at the molecular level was determined using qPCR.

#### *NPR1* gene

**Fig 7:**
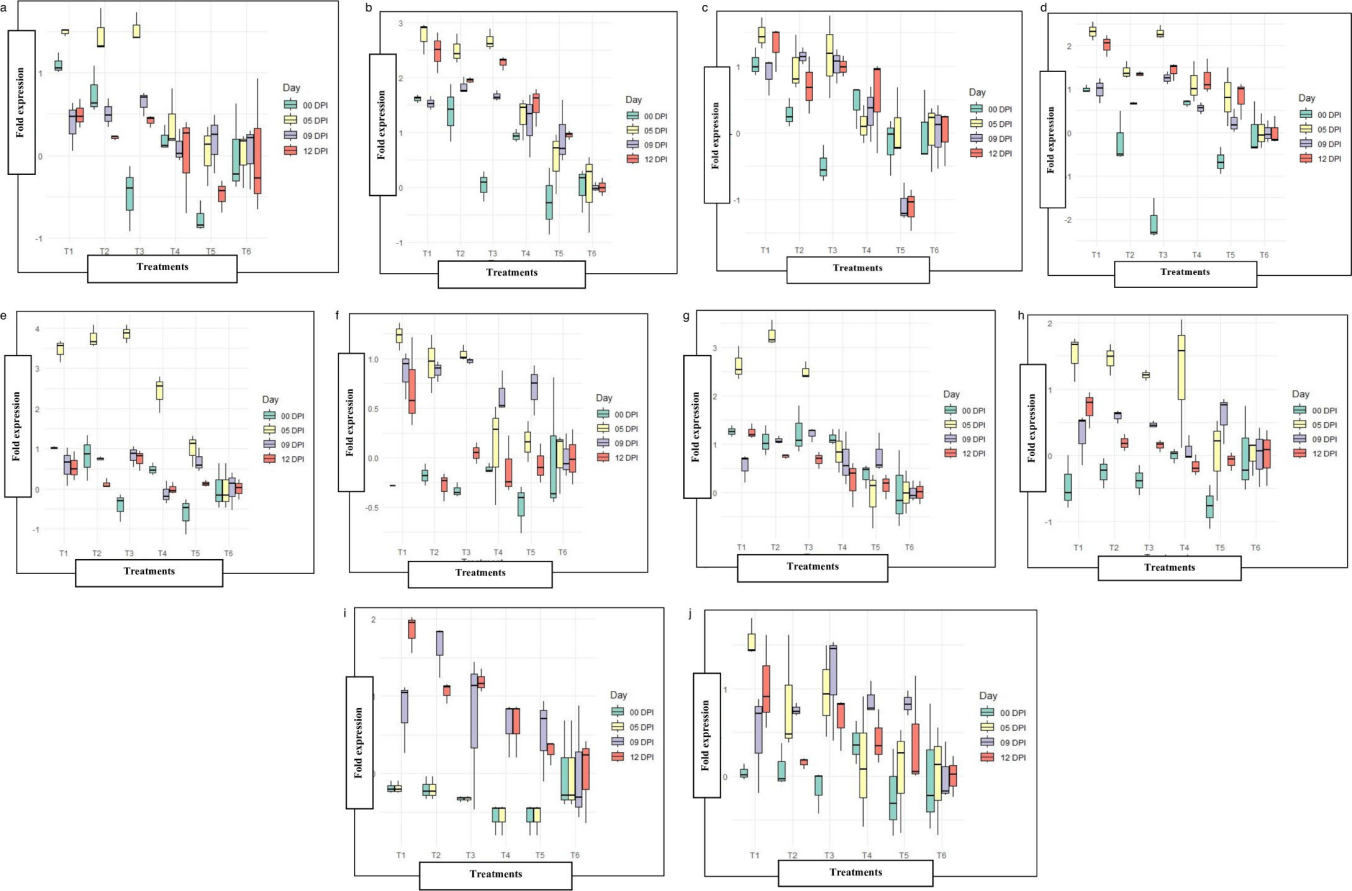
Expression pattern of defense genes: (a) *NPR1*, (**b**) *PPO*, (**c**) *WRKY33B*, (**d**) *MAPKK1*, (**e**) *PDF 1.2*, (**f**) *JAR1*, (**g**) *PR1,* (**h**) *PAL* (i) *LOX1*, and (j) *MYC2*. The expression patterns were observed at different time intervals post-inoculation with GBNV (0, 5, 9, and 12 DPI). The actin gene (ACT) was used as an internal reference. The normalized fold expression of the genes at various intervals was set to 1.0 against healthy control. *P*-value (*P* < 0.05) for the treatment effect in ANOVA analysis, indicates that at least one treatment has a significant effect on gene expression.

The gene *NPR1* is a key player in plant immunity and acts as a master regulator of the plant hormone salicylic acid (SA). The transcript level of the *NPR1* gene was highest in the tomato plants challenged with *B. melitensis* treated plants against GBNV at 5 DPI (1.49). In *B. velezensis* treated plants, the expression level of *NPR1* was downregulated at 0 DPI (0.48-fold); however, at 5 DPI, the expression level was increased by 1.52-fold. Subsequently, the transcript level declined uniformly at 9 DPI in all the treatment groups. (Fig. 7a). In untreated inoculated control, upregulation of *NPR1* (0.17-fold) was seen at 9 DPI.

#### *PPO* gene

The enzyme polyphenol oxidase catalyzes the oxidation of phenols to quinones. The PPO activity ultimately results in the production of reactive oxygen species to cope with stresses. Assessing the expression of PPO revealed an increase in transcripts in bacterial endophyte treated plants at 5 DPI. The expression level was higher in *B. melitensis* treated tomato plants (2.76- fold), which was a 2.19-fold higher, than the control treatment (0.51) at 5 DPI. The PPO expression was downregulated at 9 DPI in all the treatments (Fig. 7b).

#### *WRKY33B* gene

*WRKY33B* genes play a key role in plant defense responses to phytopathogenic organisms. They directly modulate the targeted gene or repress the other transcription factors (TFs) gene. The relative expression of transcription factor *WRKY33B* revealed, that the *WRKY33B* gene was upregulated at 0 DPI in the plant treated with the bacterial endophytes *viz*., *B. melitensis* (1.04-fold), *B. licheniformis* (0.28-fold), and *B. vallismortis* (0.44-fold). In *B. velezensis* treated plants, there was a downregulation of 0.48 fold at 0 DPI against control (0.16). The transcript level was induced to a maximum in *B. melitensis* treated plants (1.40-fold) at 5 DPI against control (0.07). The transcript level declined by 1.12-fold in inoculated control plants at 12 DPI when compared to bacterial endophyte treated plants (Fig. 7c).

#### *MAPKK1* gene

Through the process of phosphorylation, MAPKKK triggers the activation of MKK, which in turn activates MPK. It is clear that the activation of MAPK cascades forms an essential component of plant immunity. *MAPKK1* gene expression levels were compared between inoculated control and bacterial endophyte treated plants, and it was found that treatment with bacterial endophytes caused *MAPKK1* gene upregulation at 5 DPI. However, the expression level was relatively high in plants treated with *B. melitensis*, leading to a 2.3-fold increase, while the expression level was only up to 0.8-fold in untreated inoculated control plants (Fig. 7d).

#### *PDF 1.2* gene

The PDF1.2 also known as *PdfL* (plant defensin-like) genes encode cysteine-rich peptides and act as a marker for the jasmonic acid (JA) signaling pathway. Upon simultaneous inoculation, the transcript level of the *PDF 1.2* gene increased on the 5 DPI in *B. melitensis* (3.46-fold), *B. licheniformis* ( 3.76-fold), *B. velezensis* (3.86-fold), and *B. vallismortis* (2.41-fold) treated plants. The expression level of the *PDF 1.2* transcript in *B. velezensis* treated plants after 5 DPI was 2.81-fold greater than inoculated control (0.99-fold) (Fig. 7e). The transcript level gradually decreased at 9 DPI.

#### *JAR1* gene

*JAR1* gene catalyzes the formation of a biologically active jasmonate-isoleucine (JA-Ile) conjugate. The transcript level of *JAR1* increased immediately after inoculation of GBNV in both bacteria treated and untreated control plants. The expression of *JAR1* transcript was 1.2-fold higher in plants treated with *B. melitensis*, followed by *B. velezensis* (1.04-fold) at 5 DPI against control (0.16-fold). Downregulation of *JAR1* transcript was observed at 12 DPI in *B. vallismortis* (0.2-fold) and *B. licheniformis* (0.1-fold) treated tomato plants. However, on comparison of the ability of bacterial endophytes to induce *JAR1* on 12 DPI, maximum expression level was observed in tomato plants sprayed with *B. melitensis* (0.64-fold) than the other bacterial endophyte treated tomato plants and inoculated control (Fig. 7f).

#### *PR1* gene

*PR1* gene is a significant member of the *PR* gene family that enhances the immunity in plants. *PR1* is activated by salicylic acid (SA) and is a major player in the SA pathway. A relative expression of the *PR1* gene revealed that the transcripts were significantly expressed at 0 DPI, irrespective of different treatments. The highest fold increase in *PR1* was observed in tomato plants challenged with *B. licheniformis* (3.27-fold)*,* followed by *B. melitensis* (2.64-fold) and *B. velezensis* (2.5-fold), respectively at 5 DPI. The transcript level declined up to 0.08-fold in the virus-inoculated control at 5 DPI. Subsequently, it slightly upregulated by 0.75-fold at 9 DPI (Fig. 7g).

#### *PAL* gene

Phenylalanine ammonia-lyase is a key enzyme in the phenylalanine metabolism pathway known for plant growth and stress response. As anticipated, the expression level of the *PAL* gene was upregulated when challenged with GBNV. A relative fold increase up to 1.5-fold was observed in plants treated with *B. melitensis* on 5 DPI, which was 1.49-fold higher when compared to inoculated control (0.015-fold). Hitherto, irrespective of various treatments, the expression level of the *PAL* transcript was downregulated from 9 DPI (Fig. 7h).

#### *LOX1* gene

The *LOX1* gene encodes the lysyl oxidase enzyme, which is involved in the oxygenation of fatty acids to hydroperoxyl derivatives and is involved in JA pathway precursors. Inoculation of GBNV resulted in the downregulation of the *LOX1* gene in both untreated and bacterial endophyte treated tomato plants at 0 and 5 DPI. The expression level varied among the treatments at 9 DPI and 12 DPI. The maximum fold changes were observed in *B. licheniformis* (1.63-fold) treated tomato plants at 9 DPI. At 12 DPI, a 1.8-fold, increase was observed in *B. melitensis* treated tomato plants. (Fig. 7i).

#### *MYC2* gene

*MYC2* (TFs) are fundamental regulators of the jasmonate (JA) signaling in plants, which is crucial for regulating stress-responsive genes. The expression level of *MYC2* varied among the treatments upon simultaneous inoculation. At 5 DPI, a relative fold increase was observed in *B. melitensis* treated plants up to 1.5-fold, followed by *B. velezensis* (0.95-fold) against GBNV inoculated control (0.04-fold). At 9 DPI, the transcripts level was highest (1.12-fold) in tomato plants treated with *B. velezensis*. The highest level of transcript was seen in *B. melitensis* treated plant even at 12 DPI (0.72-fold), followed by *B. velezensis* (0.3-fold) in comparison with GBNV-inoculated control (Fig. 7j).

**Fig 8:**
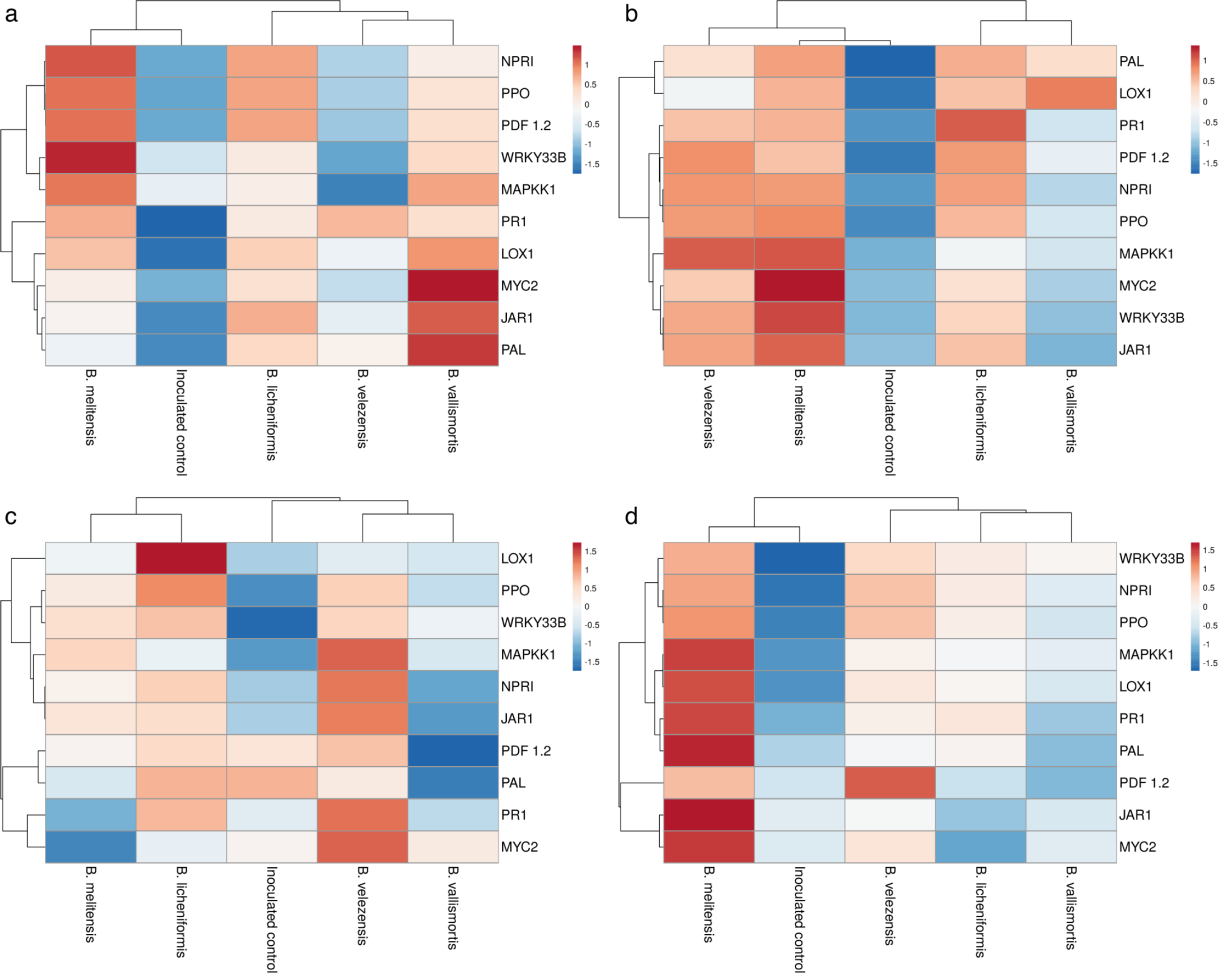
Heat map on defense gene expression during tri-trophic interaction at different DPI *viz.,* (**a**) 0 DPI, (b) 5 DPI, (**c**) 9 DPI, and (d) 12 DPI. ClustVis 2.0 software was used to generate heatmap. Red indicates high expression of a particular gene, white indicates no upregulation of a particular gene, and Blue indicates downregulation of a particular gene.

*P*-value (*P* < 0.05) for the treatment effect in ANOVA analysis indicates that there is strong evidence that at least one treatment has a significant effect on gene expression. The generation of heat map revealed the differential expression of 10 genes in bacterial endophyte-treated plants at different intervals. The expression was grouped into three subgroups. The expression of all the ten defense genes was upregulated in *B. melitensis*-treated tomato plants at 5 and 9 DPI, this was followed by *B. velezensis*. At 12 DPI, the genes were slightly downregulated in *B. melitensis* treated tomato plants (Fig. 8). On contrary to this, at 12 DPI, there was slight upregulation of defense genes ranging from 0.5 to 1.0 in *B. velezensis* treated tomato plants. Hence, these two bacteria were considered for further studies.

### Efficiency of *B. melitensis* (CNEB54) and *B. velezensis* (CNEB26) in the management of GBNV and plant growth promotion in tomato

**Fig 9:**
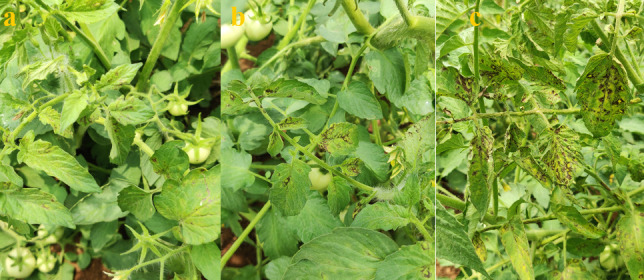
Efficacy of B. *melitensis* and *B. velezensis* in the management of GBNV in tomato. (a) Incidence of GBNV in *B. melitensis*-treated tomato plants*,* (b) incidence of GBNV in *B. velezensis*-treated tomato plants*,* and (c) incidence of GBNV in *untreated* treated tomato plants.

**Fig 10:**
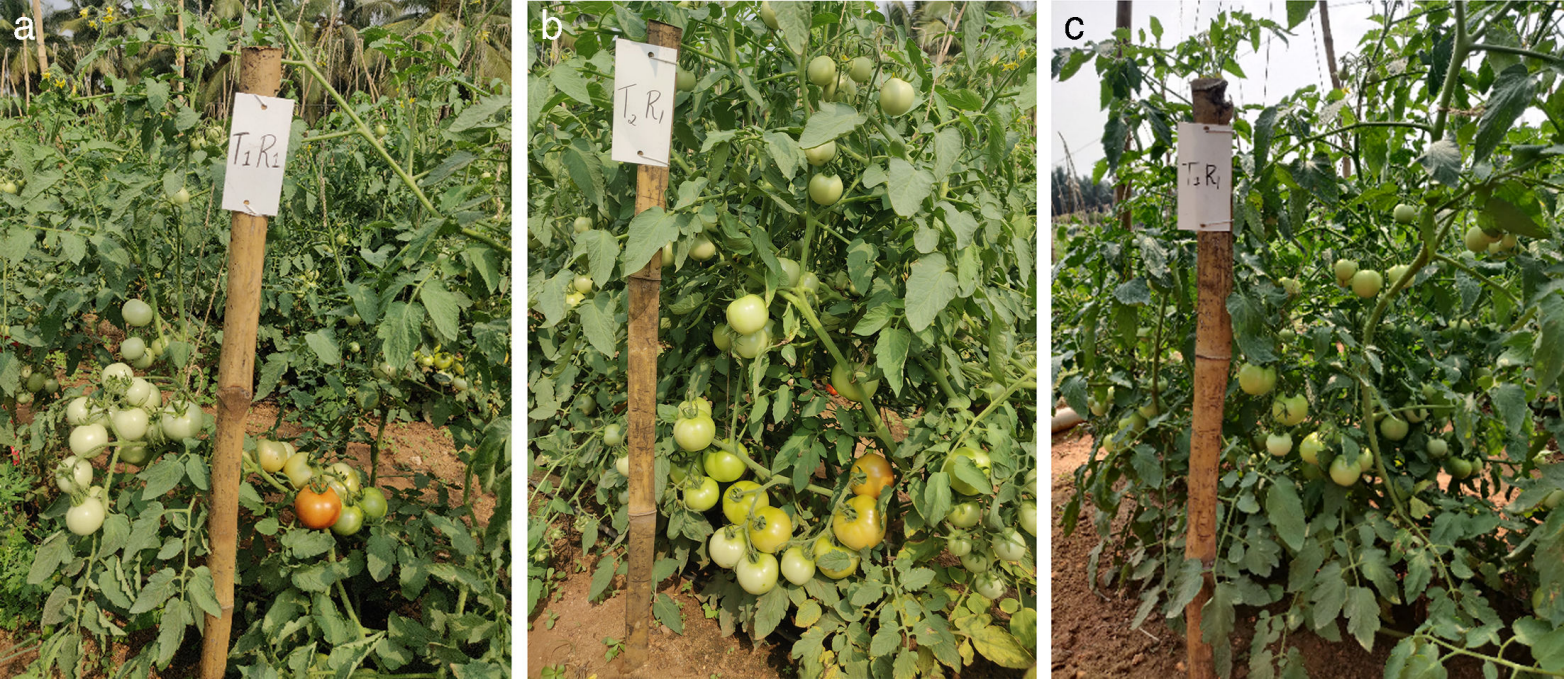
Efficacy of B. *melitensis* and *B. velezensis* on plant growth promotion in tomato. (a) *B. melitensis*-treated tomato plants, (b) *B. velezensis*-treated tomato plants, (c) *untreated* treated tomato plants.

**Fig 11:**
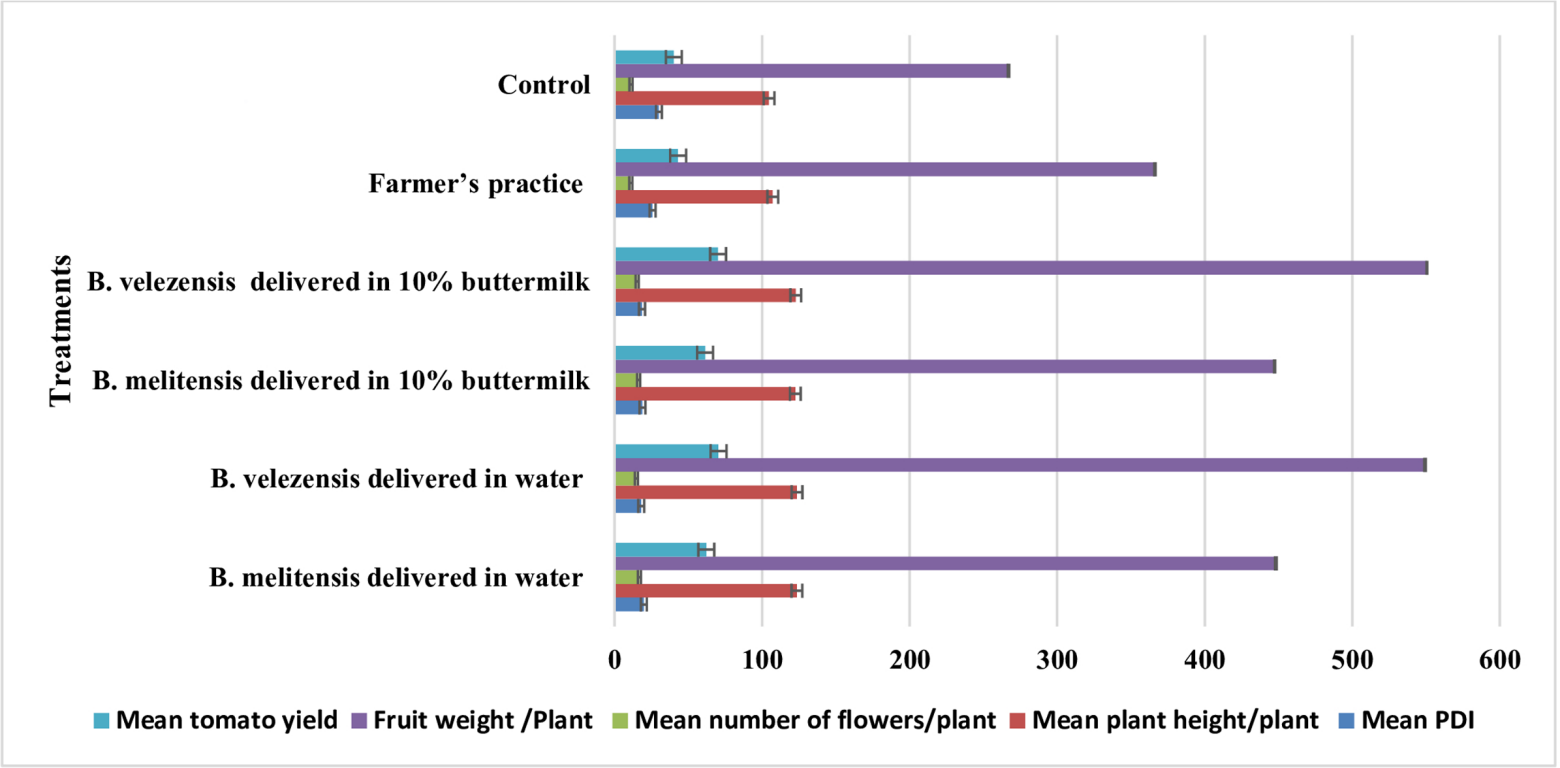
Efficiency of bacterial endophytes against GBNV and its effect on plant growth promotion. Plant growth parameters including, plant height, number of flowers, and fruits /plants were recorded in 20 plants from each replication of different treatments. Plants were selected at random and tagged to record observations on various traits. The error bar indicates the standard error obtained by three replications.

The results indicated that soil drenching and foliar application of cell suspension culture of *B. velezensis* (1.5%) delivered in water at fortnightly intervals resulted in only 18.1% GBNV incidence, when compared to 30.1% in the untreated control. This was followed by cell suspension culture of *B. velezensis* (1.5%) delivered in 10% buttermilk as soil and foliar application (18.6); cell suspension of *B. melitensis* (1.5%) delivered in 10% buttermilk (18.9%), and cell suspension culture of *B. velezensis* (1.5%) delivered in water (19.89%) (Table 4; Fig. 9). There was no significant difference between the treatments using cell suspension culture of bacteria suspended in water and buttermilk, as both performed at par in the field condition. However, the growth promotion activities were notably higher with the application of the cell suspension culture of *B. velezensis* in water. This treatment resulted in a plant height of 123.70 cm, an average of 14.87 flowers/plant, 549.3 g of fruit/plant and an anticipated yield of 70.2 ton/ha. In comparison, the untreated control recorded a plant height of 104.73 cm, 11.17 flowers per plant, a fruit weight of 267 g per plant, and an anticipated yield of 40.2 tonnes per hectare (40.2 ton/ha) (Fig. 10 and 11).

**TABLE 4:** Efficiency of bacterial endophytes against GBNV and its effect on plant growth promotion^*a*^^,^^*b*^

S. no	Treatment details	Mean PDI^*c*^	Percent reduction over control	Plant height (cm)^*d*^	Number of flowers^*e*^	Fruit weight /plant (g)	Anticipated yield (t/ha)
T1	*B. melitensis* (1.5%) delivered in water	19.89^c^ (26.49)	33.92	123.63^a^ (11.11)	16.92^a^ (24.27)	448.2^b^ (21.16)	62.2^b^ (9.86)
T2	*B. velezensis* (1.5%) delivered in water	18.1^d^ (25.3)	39.86	123.70^a^ (11.12)	14.87^c^ (22.67)	549.3^a^ (23.43)	70.5^a^ (10.29)
T3	*B. melitensis* (1.5%) delivered in 10% buttermilk	18.9^cd^ (25.91)	37.20	122.53^b^ (11.06)	16.40^ab^ (23.93)	447.4^b^ (21.15)	61.4^b^ (10.70)
T4	*B. velezensis* (1.5%) delivered in 10% buttermilk	18.64^cd^ (25.57)	38.07	122.83^b^ (11.08)	15.30b^c^ (23.02)	550.6^a^ (23.46)	70.2^a^ (10.59)
T5	Farmer’s practice	25.8^b^ (27.8)	14.28	107.27^c^ (10.35)	11.07^d^ (19.430)	366.3^c^ (19.13)	43.1^c^ (9.4)
T6	Control	30.1^a^ (31.2)	–^*f*^	104.73^d^ (10.23)	11.17^d^ (19.51)	267.0^d^ (16.33)	40.2^d^ (9.23)
	SE (d)	0.556	–	0.565	0.634	0.349	0.401

^*a*^ Plants were selected randomly and tagged to record observations on various traits.

^*b*^ The values of various triats were normalized to 0–1 scale and ANOVA was carried out.

^*c*^ Values are average of 30 plants.

^*d*^ Values in the parentheses are arcsine-transformed values.

^*e*^ In a column, means followed by a common letter are not significantly different at 5% level by DMRT.

^*f*^ “–” indicates nill.

## DISCUSSION

The GBNV infects a wide range of hosts, posing a significant threat to agricultural and horticultural crops (1, 22, 23). Symptoms of GBNV infection on tomatoes and cowpea plants have been well documented in previous studies (17, 24). Outbreaks of GBNV of up to 100% have been reported in many tomato-growing regions (3). Despite the efforts made to develop resistant cultivars for managing GBNV by plant breeders, pathologists, and entomologists, till date no resistant cultivars have been identified for managing GBNV. However, the attempts made to manage GBNV across the globe rely upon vector control and cultural practices. However, they are not effective, and the continuous application of insecticides for the management of thrips species *viz., Frankliniella schultzei, Scirtothrips dorsalis*, and *Thrips palmi* has polluted the environment. Therefore, it is imperative to develop environmentally safe management strategies to address this issue. Besides, one school of thought also emphasizes the use of plant growth-promoting rhizobacteria (PGPR) to mitigate plant pathogens including plant viruses. However, the application of PGPR to mitigate GBNV and other viruses is only in the stage of infancy (22, 25). Several studies have demonstrated the antiviral activity of specific bacterial isolates against various viruses. Furthermore, research work by earlier workers has authenticated the potential role of bacterial endophytes in the suppression of symptoms of tospoviruses. Under protected conditions, the application of *Enterobacter asburiae* BQ9 (26) induced resistance against tomato yellow leaf curl virus (TYLCV) in tomatoes and reduced the severity of the disease. Foliar application of *B. amyloliquefaciens* (14) protected *N. benthamiana* and pepper under field conditions against cucumber mosaic virus. With this background, we assessed the antiviral efficacy of 15 bacterial endophytes against GBNV. The local lesions and symptom expression of GBNV in cowpea was reduced up to 90% when challenged with *B. melitensis*, *B. velezensis*, *B. licheniformis,* and *B vallismortis* compared to untreated inoculated control. According to the study conducted by Rajamanickam and Nakkeeran (16), *Bacillus spp*. had an antiviral effect against GBNV in chilli. Reduction in lesion number and symptom expression in cowpea was up to 87.5% when challenged with *Bacillus* spp. Application of *Bacillus* consortium 24 hours prior to inoculation of CMV significantly decreased the incidence of CMV symptoms in ridge gourds (27).

**Fig 12:**
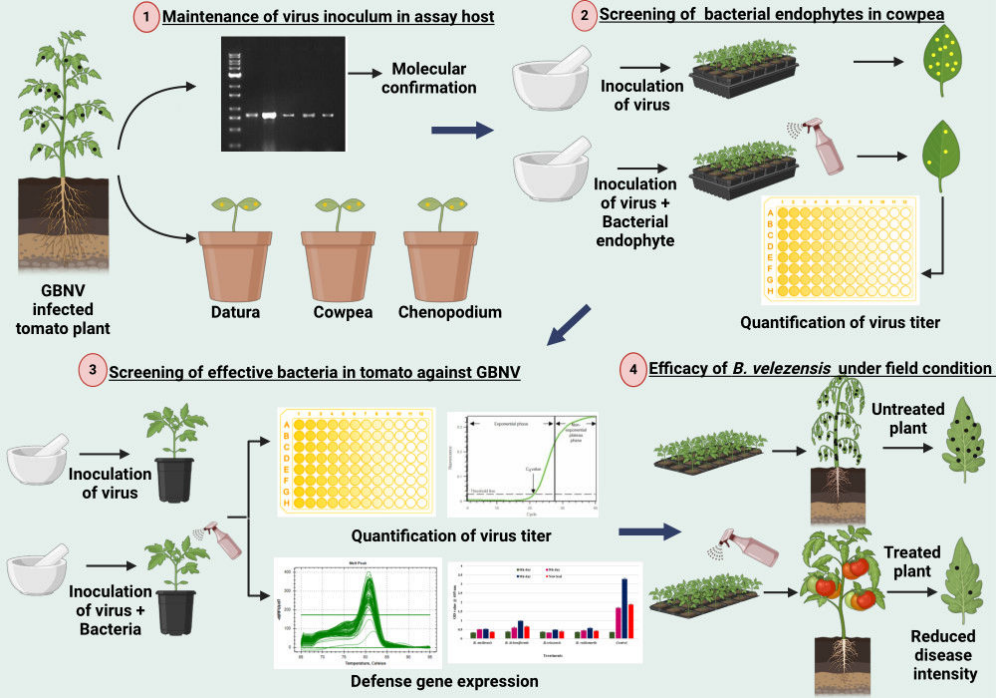
Graphical abstract of the study design.

Simultaneous inoculation of bacterial endophyte challenged with GBNV reduced the virus titer both in cowpea and tomato plants (Fig. 12). Bacterization of cowpea phylloplane with *B. melitensis* challenged with GBNV reflected that the absorbance value of GBNV was 0.210 as against untreated inoculated control (1.37). Interestingly, a delay in symptom expression was observed in endophyte-treated tomato plants. Expression of symptoms was between 11 and 13 days as the bacterial endophytes hindered the movement of the virus within tomato plants. Our findings were similar to the studies conducted by Beris et al. (28) where the tomato plants treated with *B. amyloliquefaciens* strain MBI600 and SA decreased the virus titer of TSWV and PVY. Similarly, Vanthana et al. (17) discovered that tomato plants treated with *B. amyloliquefaciens* VB7 reduced the GBNV titer and disease severity. Foliar application of *B. amyloliquefaciens* CRN9 during the early stages of chilli crop growth regulated MAMP-triggered immunity against GBNV and contributed toward the reduction of virus titer in chilli against GBNV (16). Several studies have been reported, on the reduction of virus titer against different plant viruses *viz*., banana bunchy top virus (29), cucumber mosaic virus (30), tomato mosaic virus (31), and tobacco streak virus (32) as a consequence of challenge inoculation by *Bacillus* spp. The copy number of GBNV N gene was also reduced in bacterial endophyte-treated plants when compared to untreated control at 12 DPI. A similar reduction in the virus copy number of GBNV was observed in *Bacillus*-treated tomato plants (24). The other important aspect of bioagents is the induction of innate immune response in plants against biotic stress. Hence, the expression pattern of various defense genes involved in induced systemic resistance was studied. Phenylalanine ammonia lyase (*PAL*) plays a significant role in plant growth and stress tolerance. In the present investigation, an increase in the transcript level of the *PAL* gene in the tomato plants treated with B. *melitensis, B. licheniformis, B. velezensis,* and *B. vallismortis* at 5 DPI was observed. Similarly, Vanthana et al. (18) observed that the *PAL* gene transcripts increased by threefold after 72 hours, 96 hours, and 120 hours in tomato plants treated with *B. amyloliquefaciens* challenged against GBNV. Lipoxygenase, aid in the transformation of linoleic acid into oxophytodienoic acid, which also play a key role in the biosynthesis of JA. In our study, the *LOX1* gene was upregulated by several fold on 9 and 12 DPI in *B. melitensis*-treated tomato plants challenged against GBNV. The increase in the activity of *LOX1* gene might have increased the JA pool and might have contributed toward the suppression of GBNV. According to Vanthana et al. (18), the *JAR1* gene was upregulated 24 and 48 hours after GBNV infection; this was reversed at 72 hours and then restored at 96 and 120 hours later. Frąckowiak et al. (33) showed in ToMV-infected plants, the upregulation of *LOX1* and *JAR1* gene was observed and suggested the involvement of JA pathway. Mitogen-activated protein kinase plays a pivotal role in signal transduction through the phosphorylation and de-phosphorylation of proteins with a specific gravity on serine or threonine residues. In our study, there was an upregulation of *MAPKK* in tomato plants bacterized with bacterial endophytes at 3 DPI and reached the peak on 5 DPI. Our results was in accordance with Mészáros et al. (34). He stated that *WRKY22* and *WRKY29* were activated by MAP kinase *MKK1*, which was induced during the interaction of flagellin. *WRKY33* facilitates the plants to prevent necrotrophic infection of pathogens. Since necrosis is the most common symptom of GBNV infection, it is reasonable to hypothesize that the *WRKY33B* plays a significant role in defense activation and reduction of necrosis by GBNV in tomatoes and cowpeas. In general, *WRKY33B* is known to be present at low levels in inoculated plants. But, it was significantly elevated in endophyte-treated plants. A significant increase in the transcriptional factor *CaWRKY*-619 was observed during the incompatible interaction between the tobacco mosaic virus and *Capsicum annum* (35). The transcript level of the *NPR1* gene was highest in the tomato plants challenged with *B. melitensis*-treated plants against GBNV at 5 DPI (1.49). Our results were contradictory to the study conducted by Vanthana et al. (17). Upon inoculation of GBNV, downregulation of *NPR1* gene transcript at 48 hours to 120 hours was observed, even though there was a slight increase at 24 hours. In 2019, even Rendina et al. (36). did not find a difference in *NPR1* expression after treatment with chitosan. The expression level of *PPO* and *PR1* gene was upregulated in the tomato plants treated with *B. melitensis* at 5 DPI. PPO acts as a scavenger against ROS and reactive phenolic compounds and protects the cells from damage caused by these molecules. Usually, there will be upregulation of *PPO* in virus-infected plants (37). According to the study conducted by yafei et al. (38), there was an upregulation of the *PPO* gene transcripts in oligo chitosan-treated tomato plants. *PR1* acts as a marker for SAR. In tomato crops, the role of PR1 during the infection of tomato leaf curl virus infection is well documented by Sahu et al. (39). According to Vanthana et al. (17), the *PR1* gene was upregulated up to 120 hours upon GBNV infection. Increased transcripts levels of *PR1* are often associated with many viral infections. In 2006, Whitham et al. (40) indicated the upregulation of *PR1* in many virus infections caused by Turnip vein clearing virus, Potato virus x, and Cucumber mosaic virus.

In the present study, the application of endophytes promoted the growth of tomato plants. According to Devi et al. (41), *Bacillus* spp., increased seedling germination of tomatoes, varied from 60% to 95%. In addition, the seeds treated with *B. velezensis* ERBS51 had the highest germination percentage (95%) and vigor index. Research by VinodhKumar et al. (15) and Vanthana et al. (17) demonstrated the beneficial effects of specific *Bacillus* isolates on plant growth. Application of bacterial endophytes to tomato plants challenged against plant viruses increased the shoot length, root length, vigor index, and seed germination. The production of phytohormones, enhanced nutrient availability, the synthesis of antibiotics, the decrease in ethylene, induction of defense genes, and resource competition are associated with the reduction of biotic stress (42).

### Conclusion

In the current scenario, the integrated approaches are gaining more importance, due to residual hazards of the chemical application. The present study confirmed that the application of endophytes plays a key role in growth promotion, immunity boosting, and suppression of GBNV in tomatoes. This technology will provide the added advantage of minimizing the pesticide usage and increasing productivity. In the future, the tritrophic interaction between the host, virus, and endophytes will generate an idea to identify the metabolite involved in the suppression of GBNV infecting important hosts such as tomatoes, groundnuts, and pulses.

## Data Availability

The original contributions presented in the study are included in the article/Supplementary.

## SUPPLEMENTAL MATERIAL

Supplemental material jvi.01803-24-s0001.pdf Figures S1 to S4; Tables S1 to S4.

ASM does not own the copyrights to Supplemental Material that may be linked to, or accessed through, an article. The authors have granted ASM a non-exclusive, world-wide license to publish the Supplemental Material files. Please contact the corresponding author directly for reuse.
