# Supplementary material for: Induction of innate immunity and plant growth promotion in tomato unveils the antiviral nature of bacterial endophytes against groundnut bud necrosis virus
Source: J Virol. 2024 Dec 31;99(2):e01803-24. doi: 10.1128/jvi.01803-24 (PMC11852893; doi:10.1128/jvi.01803-24)
Supplement: Supplemental material — Figures S1 to S4; Tables S1 to S4. [file jvi.01803-24-s0001.pdf]

Figure legends:

Figure S1: Maintenance of GBNV inoculum in assay hosts and in tomato. Image a-d showcase the symptom expression observed in different assay host: a. *Vigna unguiculata,* b. *Chenopodium quinoa* , c. *Nicotiana tabacum*, d. *Datura stramonium.* Image e-f represents the symptom expression in main host tomato: e. Necrotic rings on tomato leaves, f. necrotic streak on stem, g. systemic infection in tomato.

Figure S2: Agarose gel electrophoresis of RT-PCR product for Nucleocapsid gene of GBNV. M: 1 Kb marker, L1: infected sample from Viralipatti, L2: Karrupadevanpatti, L3: Thumbhichipalyam, L4: Koneripatti, L5: Positive control

Figure S3: Efficacy of bacterial endophytes against GBNV in cowpea upon pre-inoculation. Three replications and five plants per replication were maintained for each bacteria. Observations were recorded at 5 days post inoculation. Data was recorded based on total number of lesions/ leaves. T1-*B. glycinifermentans* CNEB17, T2-*B. altitudinus* BALT , T3-*B. mojavensis* CNEB 14, T4- *B. barbaricus* NPBR1, T5-*B. subtilis* YEBL5, T6-*B. albus* YEBN2, T7-*B siamensis* YEBN1, T8- *B. amyloliquefaciens* CNEB24, T9-*B. vallismortis BAVE5*, T-10 *B. endophyticus* BA453, T11-*B. melitensis* CNEB54, T12- *B. aerophilus* CNEB3, T13-*B. licheniformis* CNEB4, T14-*B. velezensis* CNEB26, T15-*B. tequilensis* NPN1, T16- untreated inoculated control.

Figure S4: Melt peak and melt curves for defense gene in qPCR assay in tomato.

Table captions

Table S1. Details of bacterial endophytes used in the study

Table S2: List of gene specific primers and their sequences used for qRT-PCR studies

Table S3. Assessing the titer value of bacterial endophytes treated cowpea plants against GBNV through DAC ELISA. The experiment was performed with biological and 3 technical replications. The samples were collected on at 5 DPI. The GBNV titer was measured at 405nm. The values after ± represents standard error obtained from three replications.

Table S4: Assessment of GBNV titer in endophytes-treated tomato plants through DAC- ELISA. The experiment was performed with three biological replications and three technical replications, for each treatment. The samples were collected on 0, 5 &10 DPI and even in newly emerged leaves. The values after ± represents standard error obtained from three replications.

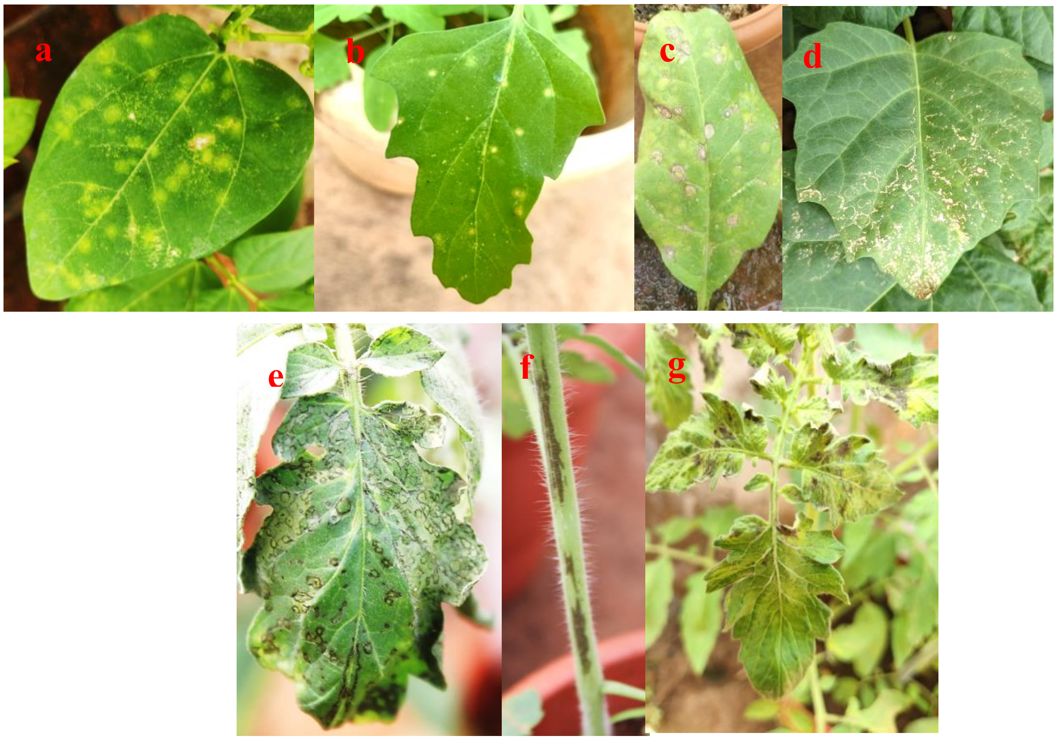

Figure S1. Maintenance of GBNV inoculum in assay hosts and in tomato

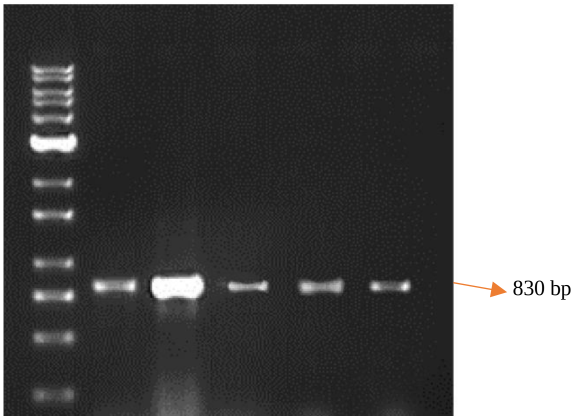

Figure S2. Agarose gel electrophoresis of RT-PCR product for Nucleocapsid gene of GBNV

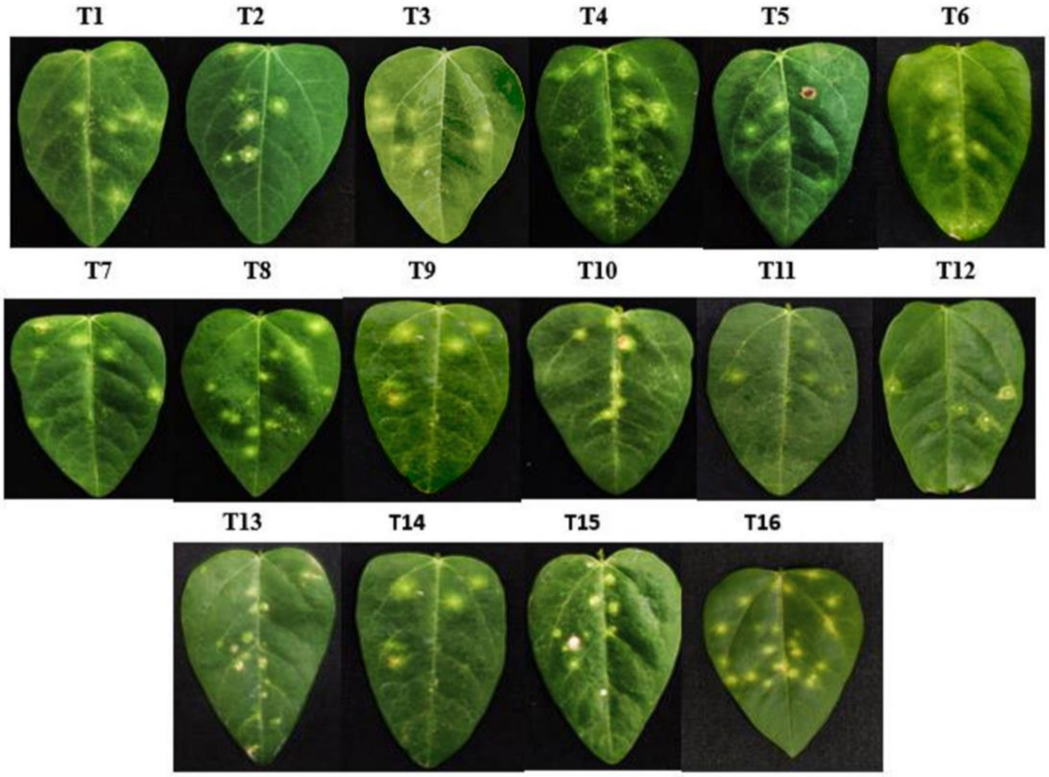

Figure S3. Efficacy of bacterial endophytes in the reduction of symptom expression in cowpea (VBN3) upon Pre- inoculation of GBNV

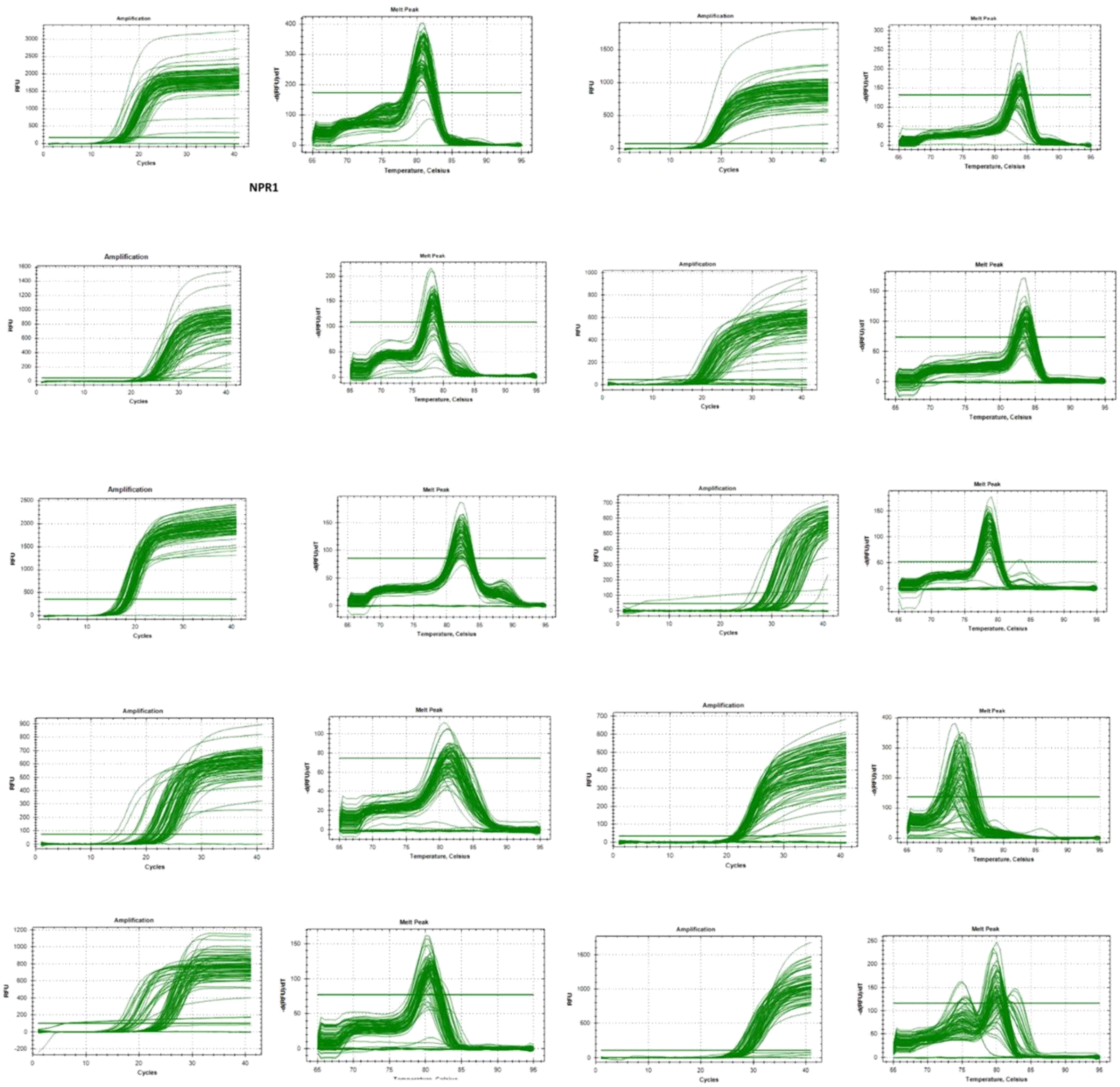

Figure S4. Melt peak and melt curves for defense gene in qPCR assay in tomato

Table S1: Details of bacterial endophytes used in the study

S.No	Bacterial endophytes	Accession numbers	Host	Isolation source
1	***Bacillus amyloliquefaciens***	**MZ485912**	Coconut	Embryo
2	***Bacillus glycinifermentans* CNEB17**	**MZ485782**	Coconut	Embryo
3	***Bacillus altitudinis* BALT**	**MT326232**	Banana	Fruit
4	***Bacillus mojavensis* CNEB14**	**MZ485478**	Coconut	Embryo
5	***Bacillus barbaricus* NPBR1**	**MT383635**	Banana	Bract
6	***Bacillus subtilis* YEBL5**	**MK259031**	Banana	Leaf
7	***Bacillus albus* YEBN2**	**MT120179**	Banana	Nectar
8	***Bacillus siamensis*YEBN1**	**MT119641**	Banana	Nectar
9	***Bacillus vallismortis* BAVE5**	**MT672715**	**Rice**	**-**
10	***Bacillus endophyticus* BA453**	**KC851835**	Rice	Grain
11	***Bacillus aerophilus* CNEB3**	**KC851834**	Rice	Grain
12	***Brucella melitensis* CNEB54**	**MN022548**	Banana	Pseudostem
13	***Bacillus licheniformis* CNEB4**	**MZ484745**	Coconut	Embryo
14	***Bacillus velezensis* CNEB26**	**MZ485916**	Coconut	Embryo
15	***Bacillus tequilensis* NPN1**	**MT383662**	Banana	Nectar

Table S2: List of gene specific primers and their sequences used for qRT-PCR studies

S.No.	Gene	Sequence	Reference
1	*MAPKK1*-F	5’ AAGCACCAGAAGGACAGAC 3’	Wu *et al*,2014b
	MAPKK1-R	5’ CCTGCGGAAGTGAAGTAAG 3’	
2	*WRKY33BB*-F	5' CCACAACAGTCTGAAATGGG 3'	Zhou *et al*, 2015
	WRKY33BB-R	5' CAGCAAAGCAATGACTCCAT 3'	
3	*NPR1*-F	5'GACCACGGCATCAAAACTCACC3'	Ekengren *et al*, 2003
	NPR1-R	5' GACTTCTTCGCTGATGCTAAGC3'	
4	*PR1*-F	5'TCAGGTGGTGTGGCGTAACTC3'	Sahu *et al*, 2012
	PR1-R	5'AAAGTACCACCACCCGTTGTTGCA3'	
5	*PAL*-F	5' GTCCTTCCTTGGGCTGCAAC 3'	López-Gresa *et al*, 2011
	PAL-R	5' GTCCTTCCTTGGGCTGCAAC 3'	
6	*PPO*-F	5' TTTGATAGGCGGAGTTTGCG 3'	Coppola 2011
	PPO-R	5' CCACCAGTTCAGTTATCGCCA 3'	
7	*LOX*-F	5' TTCATGGCCGTGGTTGACA 3'	Corrado, 2007
	LOX-R	5' AACAATCTCTGCATCTCCGG 3'	
8	*JAR1*-F	5'TCTAGAATGAAGATGATGGTGGA AAATATTG 3'	Wang and Hsieh, 2012
	JAR1-R	5'ACATTGGGACGACCGGTAAGATCT 3'	
9	*MYC*-F	5' TGGCAGCGCTCATGGAATTA 3'	Vanthana *et al*, 2022
	MYC-R	5' TCAACGCTACCCTAAGCTGC 3'	
10	*PDF 1.2*-F	5' ATCACCCTTATCTTCGCTGC3'	Bi *et al*, 2010
	PDF 1.2-R	5' TGCTGGGAAGACATAGTTGC 3'	
11	*Act*-F	5'AGGCAGGATTTGCTGGTGATGCT 3'	Mascia *et al*, 2010
	*Ac*t-R	5'ATACGCATCCTTCTGTCCCATTCCGA3'	

Table S3. Assessing the titer value of bacterial endophytes treated cowpea plants against GBNV through DAC ELISA

S.No	Treatments	OD value at 405 nm*
1	*Bacillus glycinifermentans*	0.484±0.02
2	*Bacillus albus*	0.498±0.04
3	*Bacillus barbaricus*	0.455±0.05
4	*Bacillus vallismortis*	0.435±0.07
5	*Brucella melitensis*	0.210±0.06
6	*Bacillus endophyticus*	0.553±0.07
7	*Bacillus velezensis*	0.361±0.04
8	*Bacillus licheniformis*	0.427±0.05
9	Inoculated control	0.837±0.01
10	Positive control	1.340±0.1
11	Healthy control	0.147±0.02

Table S4. Assessment of GBNV in endophytes-treated tomato plants through DAC ELISA

Treatments	0th day	5th day	9th day	New leaf
Brucella melitensis	0.317±0.02	0.495±0.01	0.533±0.03	0.369±0.01
Bacillus licheniformis	0.374±0.03	0.593±0.06	1.673±0.02	0.915±0.03
Bacillus velezensis	0.362±0.03	0.323±0.01	0.481±0.02	0.395±0.04
Bacillus vallismortis	0.353±0.02	0.431±0.01	0.641±0.05	0.426±0.02
**Untreated control**	0.349±0.01	1.72±0.03	3.368±0.07	1.884±0.04
**Healthy control**	0.332±0.02	-	-	-
